# Evaluating the relationship between antidrug antibodies and efficacy and safety outcomes in patients with Fabry disease receiving enzyme replacement therapy: a systematic literature review

**DOI:** 10.1186/s13023-026-04438-8

**Published:** 2026-07-16

**Authors:** Patricio Aguiar, John A. Bernat, Derralynn Hughes

**Affiliations:** 1 Inborn Errors of Metabolism Reference Center, Unidade Local de Saúde de Santa Maria, Lisbon, Portugal; 2 Faculty of Medicine, Lisbon University, Lisbon, Portugal; 3 University of Iowa Health Care, Iowa City, IA USA; 4 Lysosomal Disorders Unit, Royal Free London NHS Foundation Trust and University College London, London, UK

**Keywords:** Agalsidase alfa, Agalsidase beta, Antidrug antibody, Disease-related biomarker, Efficacy, Enzyme replacement therapy, Fabry disease, Pegunigalsidase alfa, Safety, Systematic literature review

## Abstract

**Background:**

Fabry disease is a lysosomal storage disorder caused by deficient activity of the enzyme α-galactosidase A, resulting in progressive accumulation of globotriaosylceramide (Gb3) and widespread tissue and organ damage. Enzyme replacement therapy (ERT) can slow disease progression but may elicit antidrug antibody (ADA) responses. The impact of ADA status on long-term ERT efficacy and safety remains incompletely understood.

**Objective:**

We conducted a systematic literature review (SLR) to examine the impact of ADA status on outcomes in patients with Fabry disease receiving ERT. We aimed to evaluate associations between ADA status and specific outcomes, and to identify remaining evidence needs.

**Methods:**

Systematic searches were conducted in MEDLINE and Embase (up to March 4, 2025), supplemented by searches of the Cochrane Library and clinical trial registries. Eligible publications reported data on the relationship between ADA status and Fabry disease-related biomarkers (Gb3, globotriaosylsphingosine [lyso-Gb3]), efficacy (renal and cardiovascular) outcomes, and safety (adverse events [AEs] and infusion-related reactions [IRRs]) outcomes.

**Results:**

Of 1763 articles screened, 24 were included (19 reported disease-related biomarkers, 10 renal outcomes, 4 cardiovascular, and 9 AE/IRR data). Most analyses of disease-related biomarkers (17/18) reported statistically significant positive associations (*p* < 0.05) between ADA positivity and elevated biomarkers: plasma lyso-Gb3 (4/8 studies), plasma Gb3 (1/3 studies), and urine Gb3 (6/6 studies). Some renal data sets (2/9) reported evidence suggesting a potential association between more rapid decline in estimated glomerular filtration rate in ADA-positive (vs ADA-negative) patients. Four out of 9 studies reported statistically significant associations between ADA positivity and IRR occurrence; most studies stratifying IRR rates by ADA status (5/7) reported higher rates among ADA-positive (vs ADA-negative) subgroups.

**Conclusion:**

This SLR found evidence that ADA positivity may be associated with increased IRR risk and may have a negative effect on some measures of ERT efficacy. Inconsistencies in the identified data were likely driven by study design differences, including prior ERT exposure, follow-up time, and ADA assessment protocols. Additional prospective studies with standardized ADA assessments and accounting for patient baseline characteristics and disease severity, are needed to better understand the clinical implications of ADA formation on ERT outcomes, including an assessment of causality.

**Supplementary Information:**

The online version contains supplementary material available at 10.1186/s13023-026-04438-8.

## Background

Fabry disease (FD) is a rare, X-linked lysosomal disorder caused by pathogenic variants in the *GLA* gene, which encodes the lysosomal enzyme α-galactosidase A (α-Gal A) [1]. These genetic alterations result in deficient or absent α-Gal A activity, leading to progressive accumulation of globotriaosylceramide (Gb3) and its deacylated derivative globotriaosylsphingosine (lyso-Gb3) in various tissues and organs, resulting in life-threatening health complications such as renal failure, cardiac rhythm disturbances, heart failure, stroke, and significant impact on quality of life [1].

Standard of care treatment for Fabry disease involves enzyme replacement therapy (ERT) in the form of infusions with a recombinant α-Gal A to counter its deficiency, or oral chaperone therapy for patients with amenable variants [1]. ERT supplements the deficient α-Gal A enzyme in order to reduce substrate accumulation and slow disease progression, thus helping to reduce clinical manifestations and improve quality of life [1]. Three options are currently indicated for patients with Fabry disease: agalsidase beta (approved in Europe in 2001 and in the USA in 2003) [2, 3]; agalsidase alfa (approved in Europe in 2001) [4], and pegunigalsidase alfa (approved in both Europe and the USA in 2023) [5, 6]. Biosimilar products are also available in some jurisdictions [7]. However, the efficacy and tolerability of ERT may be affected by the development of antidrug antibodies (ADAs), a recognized challenge with biologic therapies [8]. ADAs directed against ERTs may alter their pharmacokinetic and pharmacodynamic properties, potentially diminishing efficacy and increasing the risk of adverse events (AEs), including hypersensitivity and/or IRR reactions [9]. The incidence of ADAs is influenced by factors such as patient sex, phenotype, *GLA* gene variant type, residual enzymatic activity, and prior ERT exposure [10–12]. The clinical impact of ADAs may also be influenced by several immunologic parameters, such as antibody titer, neutralizing capacity, binding affinity and avidity, as well as other patient-specific factors [10, 11, 13].

ADAs can be classified into two main groups: neutralizing ADAs, which inhibit enzymatic activity or block receptor-mediated uptake (typically IgG1 and IgG4 subtypes), and non-neutralizing ADAs, which bind the enzyme without directly inhibiting its function [9]. Although non-neutralizing ADAs do not directly affect enzyme activity, they may still alter the pharmacokinetics of ERTs and promote the formation of immune complexes; these immune complexes may be cleared by macrophages, potentially reducing drug bioavailability and triggering inflammatory responses [9].

Current evidence suggests that ADA development is most common in men with classic Fabry disease, who produce no endogenous α-Gal A [9]. In addition, nonsense or frameshift mutations in the *GLA* gene, higher baseline lyso-Gb3 levels, and initial treatment with agalsidase beta were also potential risk factors identified by a predictive modeling analysis [14].

Determining causal relationships with respect to ADA status is challenging. Understanding causal relationships between patient characteristics and ADA development is hampered by methodological limitations within the current evidence (e.g. lack of ADA assay standardization, infrequent differentiation of ADA subtypes, and heterogeneity of evaluated outcomes), and determining causality between ADA status and clinical outcomes is complicated by biological and pathophysiological factors (e.g. progressive refinement of antibody specificity through somatic hypermutation and affinity maturation, and the association between the classic phenotype and more severe disease) [11, 15]. We conducted a systematic literature review (SLR) to consolidate available evidence from studies that report empirical data on the association between ADAs and measures of ERT efficacy and safety in patients with Fabry disease. Our aim was to evaluate whether associations exist between ADA status and specific outcomes, and to understand remaining evidence needs.

## Methods

### Search strategy

This SLR was conducted in accordance with the PRISMA guidelines. Systematic searches were conducted of the electronic bibliographic databases MEDLINE and MEDLINE In-Process (via PubMed), Embase (via Elsevier), and the Cochrane Library (CENTRAL and CDSR). Database-specific search strings and limits are provided in Supplementary Tables S1–S3. To identify additional records of potential relevance, manual complementary searches were conducted of professional organization websites for non-indexed congress abstracts, clinical trial registries, and health technology organization websites (Supplementary Table 4), and of the reference list of recent, topic-relevant, SLRs and meta-analyses (Supplementary Table 5). Searches were conducted on January 12, 2024 with no date restrictions (limited to English language publications); MEDLINE and Embase searches were updated on March 4, 2025.

### Study selection

Articles were included if they met pre-specified eligibility criteria. Eligible publications were full-text articles (no time limit) or abstracts (from the prior two years) reporting pre-specified efficacy and safety outcomes, stratified by ADA status for patients with Fabry disease receiving ERT in any of the following clinical contexts: randomized controlled trials (RCTs); single-arm studies; prospective observational studies; retrospective studies; registry analyses; database analyses; *post hoc* analyses; pooled analyses; case series (including ≥ 2 but ≤10 patients); systematic reviews (including meta-analyses); epidemiological and/or ecological studies; and prognostic studies. ADA status was pre-specified as reports of ADA levels (titers, concentration) or presence of ADAs, inhibition of agalsidase alfa or beta, or pegunigalsidase alfa, ERT inhibition, neutralizing ADAs, or serum-mediated ERT inhibition. Outcomes of interest for the review were pre-specified as: renal outcomes/events; cardiovascular disease/outcomes; disease-related biomarkers (Gb3 levels, lysoGb3 levels), and safety/AE/IRR outcomes. Articles reporting single-person case reports, animal and pre-clinical studies, consensus reports, non-systematic review articles, or survey data were excluded; non-English language articles were also excluded.

Study selection followed a two-stage screening process, involving initial screening of titles and abstracts to exclude clearly irrelevant studies, followed by full-text review of potentially relevant articles to determine final eligibility. Articles were screened by two independent researchers; disagreements were resolved by a third adjudicating researcher. Full details of the inclusion and exclusion criteria applied at each screening stage are provided in Supplementary Table 6. To ensure the literature included was up to date, an extension PubMed search was performed for March 2025–March 2026; however, no additional studies met the inclusion criteria.

### Quality assessments and data extraction

Quality/risk of bias assessment of RCTs was conducted to standards recommended by the UK National Institute for Health and Care Excellence [16]. Non-randomized studies were assessed using the validated Critical Appraisal Skills Programme (CASP) checklist [17]. For the purposes of this review, non-randomized studies were designated as ‘prospective’ if both the ADA and outcome assessments were conducted prospectively, and as ‘retrospective’ if either one (or both) of these parameters was assessed retrospectively.

Additional aspects of study design relevant to the interpretation of the reported relationship between ADA status and clinical and/or disease-related biomarker outcomes (e.g. randomization by baseline ADA status, statistical adjustments for confounders) were also explored during manuscript development. Data were extracted by one reviewer and quality checked by a second reviewer; any differences were reconciled between the two reviewers or by a third reviewer.

### Data synthesis

Owing to substantial heterogeneity in study designs, ADA assessment methods, and outcome measures across the literature, a formal meta-analysis was not feasible. Instead, a rigorous exploratory descriptive and narrative synthesis was undertaken to identify key trends, patterns, and knowledge gaps in the reported associations between ADA status and Fabry disease-related biomarkers, and in ERT efficacy and safety outcomes.

Graphical panels were developed for each outcome type (efficacy [disease-related biomarkers, renal, cardiovascular] and safety/tolerability) to summarize the presence/absence of significant associations with ADAs across different studies and outcome assessments. A reported *p*-value of ≤0.05 was considered to indicate a statistically significant association (a *p*-value of ≤0.10 was considered to indicate a statistical trend). The term ‘numerical trend’ is used in the text (but not in the summary panels) to describe data trends in the direction of a positive association that had a *p-*value of >0.10 or that was reported without a stated test of association for the comparison. This approach was taken to allow a broader qualitative synthesis in this rare disease with limited literature. A study was deemed to show a significant positive association if the positive association occurred in at least one subgroup (e.g. men or women; ERT treatment type) or, assuming no significant, contradictory findings were observed between subgroups or time points. For outcomes for which the change from baseline was assessed but a statistical test comparing ADA-positive and ADA-negative groups was not reported, an association was still considered present if a significant change from baseline for the outcome of interest was reported in one ADA subgroup but not the other, provided there were no significant, contradictory findings between subgroups or time points. Studies reporting no positive association for an outcome or reporting numerical data without a test/statement of association, were indicated as showing no positive association in summary panels; however, such studies are distinguished from each other when described in the following text.

For the analysis of IRR by ADA status, weighted mean proportions of ADA-positive and ADA-negative patients with IRRs were calculated for the pooled population included in the review. For the ADA-positive population, the weighted mean was calculated by weighting the percentage of ADA-positive patients with IRRs in each study by that study’s contribution to the total ADA-positive population across all studies included in the review. Similarly, the weighted mean for the ADA-negative population was calculated by weighting the percentage of ADA-negative patients with IRRs in each study by that study’s contribution to the total ADA-negative population across all studies in the review.

## Results

### Search results and study characteristics

**Fig. 1:**
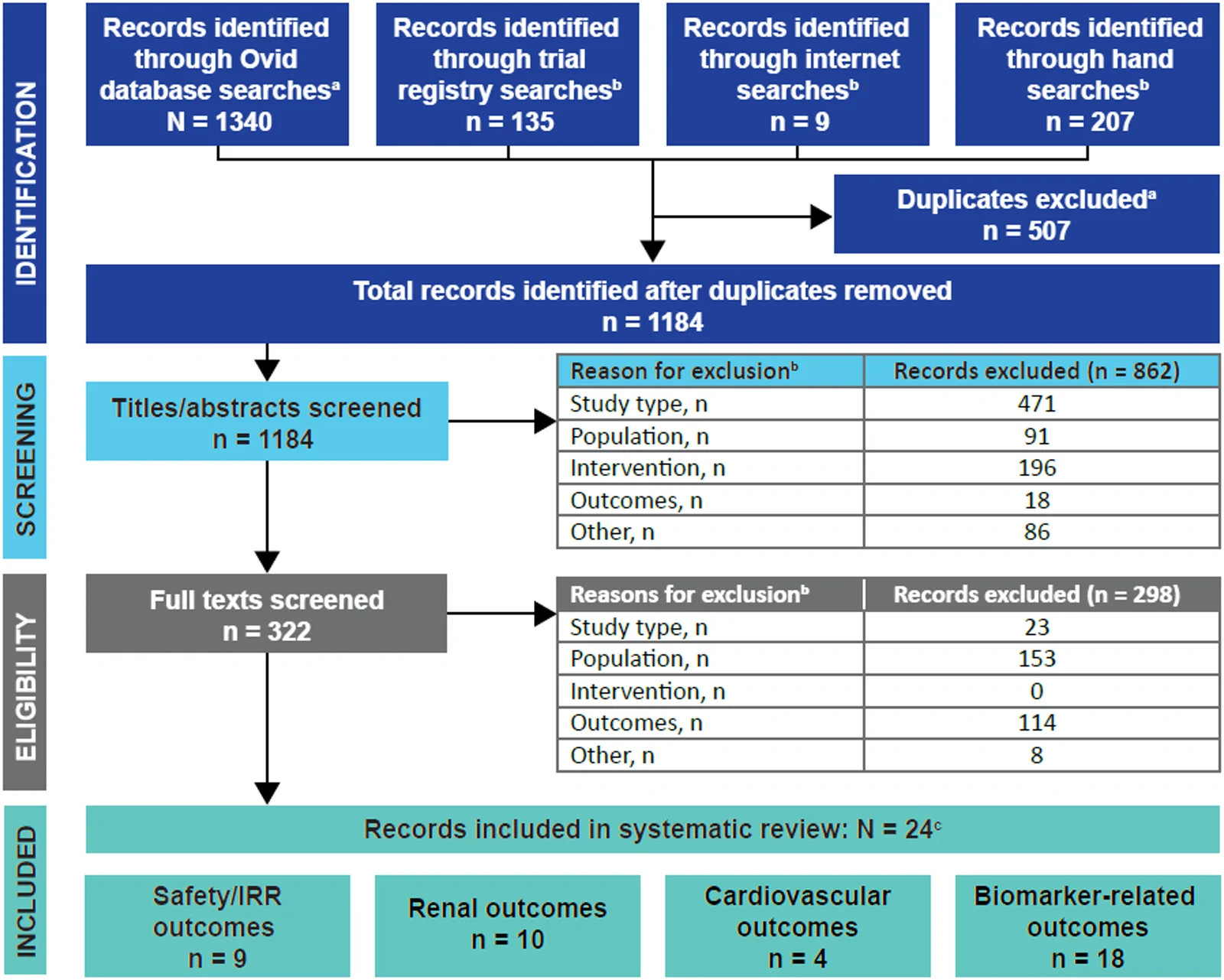
PRISMA diagram. ^a^Defined as any paper that analyzed data from the same study as a primary paper but that did not include any additional relevant data. ^b^Did not meet the following pre‑specified eligibility criteria: (1) population: patients with Fabry disease; (2) intervention: ERT; (3) study type: randomized, controlled, prospective clinical trials; single-arm studies; prospective observational studies; retrospective studies; registry analyses, database analyses, post hoc analyses, pooled analyses, case series (including ≥ 2 but ≤10 patients), systematic reviews (including meta-analyses), epidemiological and/or ecological studies, prognostic studies; (4) outcomes: reported levels (titers, concentration) or presence of: ADAs, inhibition of agalsidase alfa or beta, or pegunigalsidase alfa, ERT inhibition, neutralizing ADAs, serum-mediated ERT inhibition and ≥1 of the following outcomes of interest: renal outcomes/events; cardiovascular disease/outcomes; disease-related biomarkers (Gb3 levels, lysoGb3 levels); safety/AE/IRR outcomes; (5) language: English language; (6) date: no limit (full-text articles), prior 2 years (abstracts). ^c^Includes articles that reported more than one type of outcome (e.g. disease-related biomarker-related outcomes and cardiovascular outcomes). ADA, antidrug antibody; AE, adverse event; ERT, enzyme-replacement therapy; Gb3, globotriaosylceramide; IRR, infusion-related reaction; lysoGb3, globotriaosylsphingosine

A PRISMA flow diagram of the search strategy is presented in Fig. 1. Of 1763 articles that were retrieved (up to March 4, 2025) and screened from the MEDLINE and Embase searches, 24 studies (no abstracts) were included [10, 11, 13, 18–38]. Characteristics of the included studies are provided in Table 1. Two were RCTs [19, 20], four were open-label single-arm trials [32, 33, 35, 36], four were prospective studies [23, 25, 34, 37], and 11 were retrospective studies [10, 11, 13, 18, 22, 24, 27–29, 31, 38]. Three studies did not follow a formal study design: one was part-comparative/part-switch study that also included some patients from a separate head-to-head study; the second included multiple patient subgroups from different studies, with differing sampling time points, interventions, and comparators, and with no pre-defined hypothesis; and the third was a retrospective analysis of a single treatment cohort but that the authors explicitly described as having ‘no formal study design’ [21, 26, 30]. Quality assessments of the included RCTs and the non-randomized, non-controlled studies are provided in Supplementary Tables 7 and 8, respectively.

**Table 1:** Characteristics of included studies

Study			Baseline characteristics			Study Tx	ADA assessment		Outcome assessment	
REF	Design	N	Demographics	Previous ERT use	% Ab		Type	Data collection/ definitions	Type	Data collection/ definitions
**Holida 2025 [**36**]**	Open-label, single-arm trial (BRIGHT; phase 3; international)	30	ADA-positive (*n* = 10) Mean age: 33.2 y Men: %NR Time since diagnosis: NR REA: NR Classic FD: NR Genotype: NR ADA-negative (*n* = 20) Mean age: 44.1 y Men: %NR Time since diagnosis: NR REA: NR Classic FD: NR Genotype: NR	ADA-positive (*n* = 10) AB 1.0 mg/kg 100% ADA-negative (*n* = 20) AA 0.2 mg/kg; 35% AB 1.0 mg/kg; 65%	ADA (PA): 67%	PA: 2.0 mg/kg every 4 w for 1 y	ERT-IgG ERTi (NAb)	Prospectively assessed (frequency NR); defined as ERT-IgG-positive if detected at baseline; of 10 patients who were ERT-IgG-positive at baseline, 6 remained positive until end of Tx, 2 became negative, and 1 was negative at all post-baseline time points (1 withdrew consent); no patients who were ERT-IgG-negative at baseline developed them during Tx; ERT-IgG were NAbs at ‘most’ time points Outcomes were assessed by ERT-IgG status only	**1.** Disease-related biomarkers (plasma lyso-Gb3) **2.** Renal (eGFR)	**1.** Prospectively assessed at baseline, and at 12, 24, 40, and 52 w of Tx **2.** Prospectively assessed every 4 w
**Wallace 2024 [**19**]**	RCT (BALANCE; phase 3; international)	77	Mean age: 44.3 y Men: 61% Time since diagnosis: NR REA: NR Classic FD: NR Genotype: NR	AB: 100% (mean duration: 5.8 y)	ADA: 34% NAb: 31%	PA 1.0 mg/kg for 2 y: 68% AB 1.0 mg/kg for 2 y: 32%	ERT-IgG ERTi (NAb)	Prospectively assessed (timing/frequency NR); defined as ERT-IgG-positive if detected at baseline; of 26 patients who had ERT-IgG at baseline, 24 (92.3%) were also NAb-positive. Outcomes were assessed by ERT-IgG status only	**2.** Renal (eGFR; men) **5.** IRRs (men)	**2.** Prospectively assessed at least monthly (including baseline) for 30 Tx visits **5.** Prospectively assessed throughout Tx; defined as any AE occurring within 2 h of infusion and deemed possibly/probably related to Tx by the investigator
**Linhart 2023 [**33**]**	Open-label, single-arm trial (BRIDGE; phase 3; international)	22	Mean age: 44.0 y Men: 68% Time since diagnosis: NR Mean REA (leukocytes): 12% of normal laboratory mean Mean REA (plasma): 11% of normal laboratory mean Classic FD: NR Genotype: NR	AA ≥ 2 y: 100% Mean ERT start age: 34.8 y	ADA: 9.1%	PA 1.0 mg/kg for 1 y	ERT-IgG ERTi (NAb)	Prospectively assessed post-Tx (timing/frequency NR); defined as ERT-IgG-positive if detected ≥ 1 during Tx; of 7 ERT-IgG-positive patients, 2 had pre-existing and NAb-positive ERT-IgG while 5 developed ERT-IgG during Tx (3 persistent; 2 transient; none NAb-positive). Outcomes were assessed by ERT-IgG status only	**2.** Renal (eGFR) **4.** AEs (HRs) **5.** IRRs	**2.** Prospectively assessed at baseline (pre-infusion) and every 4 w during Tx **4.** Prospectively assessed throughout Tx (primary study objective) **5.** Prospectively assessed throughout Tx and defined as any AE occurring within 2 h of infusion and deemed possibly/probably related to Tx by the investigator
**van der Veen 2022 [**23**]**	Prospective (FIELD study; Netherlands, Norway)	7	Mean age: NR (pediatric at ERT start) Men: 100% Time since diagnosis: NR REA: NR Classic FD: 100% Genotype: 43% missense	NR	NR	AA 0.2 mg/kg for ⁓10 y (*n* = 3) AB 1.0 mg/kg for ⁓10 y (*n* = 4)	ERTi (NAbs)	Prospectively assessed during Tx (timing/frequency NR); defined as ERTi-positive if ERTi titer (resulting in >50% ERTi) was > 6; 10 out of 23 ERTi-positive patients became ERTi-negative or alternated between positive and negative status during Tx	**1.** Disease-related biomarkers (plasma lyso-Gb3)	Prospectively assessed during Tx (timing/frequency NR)
**Khan 2021 [**35**]**	Open-label, single-arm trial (phase 3/4; Canada)	167	ADA-positive: 7.2% Mean age: 48.9 y Men: 55% Time since diagnosis: NR REA: NR Genotype: NR	AA RB: 46% Other: 28% None: 26% AB: 1.2%	ADA: 7.2%	AA AF 2.0 mg/kg AA AF 0.2 mg/kg Mean number of infusions: 116	ERT-IgG ERTi (NAbs)	Prospectively assessed during Tx (timing/frequency NR); defined as ERT-IgG (or NAb)-positive if ERT-IgG (NAb) present at ≥ 1 time point; of 42 patients with ERT-IgG, 27 (64.3%) had ≥ 1 NAb-positive result, 29 (69.0%) had persisting ERT-IgG, 16 (38.1%) had persisting NAb, and 5 (11.9%) had transient NAb Outcomes were assessed by ERT-IgG status only	**5.** IRRs	Prospectively assessed throughout Tx; reaction defined as infusion-related if it occurred within 12 h of infusion and deemed possibly/probably related to Tx by the investigator
**Lenders 2021 [**34**]**	Prospective (clinical records; Germany)	78	Switch group (*n* = 22) Mean age: 40 y Men: 64% Time since diagnosis: NR REA: NR Classic FD: NR Genotype: NR Re-switch group (*n* = 39) Mean age: 48 y Men: 56% Time since diagnosis: NR REA: NR Classic FD: NR Genotype: NR Regular dose group (*n* = 17) Mean age: 42 y Men: 77% Time since diagnosis: NR REA: NR Classic FD: NR Genotype: NR	AB 1.0 mg/kg for ≥1 y	Switch group: 45.5% (5/11) Re-switch group: 33.3% (7/21) Regular dose group: 50.0% (4/8)	Switch group: AA 0.2 mg/kg, or dose-reduced to AB 0.3 then switched to AA 0.2 mg/kg Re-switch group: AB 0.3 mg/kg or AA 0.2 mg/kg for ≥24 mo then re-switched to AB 1.0 mg/kg Regular dose group: continued on AB 1.0 mg/kg	Unclear (NR)	Prospectively assessed (frequency/timing NR)	**1.** Disease-related biomarkers (lyso-Gb3)	Prospectively assessed at baseline, 38 mo (switch and re-switch) and 88 mo (regular dose group)
**Riccio 2021 [**29**]**	Retrospective (clinical records; Italy)	56	ERT-experienced (n = 11) Mean age: 50.4 y Men: 64% Time since diagnosis: NR Mean REA: 0.96 mmol/h/mL Classic FD: 100% Genotype: NR ERT-naive (n = 42) Mean age: 45.8 y Men: 50% Time since diagnosis: NR Mean REA: 2.36 mmol/h/mL Classic FD: 64% Genotype: NR	ERT-naive: 79%	NR	Stepwise infusion rate reduction protocol: 19 infusions (AB 50–80 mg) or 22 infusions (AB 80–105 mg)	ERT-IgG	Retrospectively assessed (clinical records); defined as ERT-IgG-positive if detected at end of infusion protocol	**5.** IRRs (ERT-naive patients)	Retrospectively assessed (clinical records at time of inclusion) for Tx period
**Tsurumi 2021 [**25**]**	Prospective (two PMS studies; Japan)	307	Mean age at ERT start: 39.5 y Men: 66% Time since diagnosis: NR REA: NR Classic FD: 62% (117/188) of patients with ERT-IgG data available Genotype: NR	24.4% (46/188) of patients with available ERT-IgG data had previously used ERT	NR	AB 1.0 mg/kg (duration (mean) [SD]: 83.6 [50.4] doses)	ERT-IgG	Prospectively assessed every 6 mo; definition of ERT-IgG-positive status NR Outcomes were assessed by ERT-IgG status only	**4**. AEs (including SAEs, ADRs, SADRs, HRs, SHRs) **5.** IRRs (including SIRRs and SIRR CRs)	**4.** Prospectively assessed every 6 mo; defined as any untoward medical event, regardless of whether a causal relationship was confirmed **5.** Prospectively assessed every 6 mo; defined as any AE occurring during infusion or post-infusion that day
**Tsukimura 2020 [**26**]**	Study design NR (Japan)	35	Mean age: NR Men: 100% Time since diagnosis: NR REA: NR Classic FD: 67% Genotype: 8 of 19 (42%) classic FD males with available data had nonsense mutations (same for missense)	NR	NR	Classic FD males (n = 35) AA 0.2 (*n* = 27) AB 1.0 (*n* = 7)	ERT-IgG ERTi (NAbs)	ERT-IgG and ERTi assessed at 6 mo; of 22 patients who were ERT-IgG-positive at 6 mo (classic FD: *n* = 20; late onset FD: *n* = 3), 20 (91%) had NAbs Outcomes were assessed by ERT-IgG status only	**1.** Disease-related biomarkers (classic FD men: plasma lyso-Gb3)	Assessed at baseline, and 6 and 24 mo
**Van der Veen 2019 [**22**]**	Retrospective (clinical records; Netherlands)	39	ERTi-positive (*n* = 23) Mean age at ERT start: 35.4 y Men: 100% Time since diagnosis: NR REA: 1.1% Classic FD: 100% Genotype: 61% nonsense/frameshift ERTi-positive (*n* = 16) Mean age at ERT start: 20.1 y Men: 100% Time since diagnosis: NR REA: 0.3% Classic FD: 100% Genotype: 19% nonsense/frameshift	NR	NR	ERTi-positive (*n* = 23) AA: 0% AB: 57% AA+AB: 44% Mean dose per infusion: 0.7 mg/kg ERTi-negative (*n* = 16) AA: 38% AB: 13% AA+AB: 50% Mean dose per infusion: 0.7 mg/kg Mean follow-up: 10 y	ERTi	Retrospectively assessed; baseline and every 6 mo; defined as ERTi-positive if ERTi titer (resulting in >50% ERTi) was > 6 at any point during Tx	**1.** Disease-related biomarkers (plasma lyso-Gb3) **2.** Renal (eGFR)	Retrospectively assessed (clinical records); baseline and every 6 mo
**Arends 2018 [**38**]**	Retrospective (international)	387	Mean age at ERT start: 46 y AA 0.2 (*n* = 248) Mean age at start of ERT: 46 y Men: 47% Time since diagnosis: X REA: X Classic FD: 66% Genotype: X AB 1.0 (*n* = 139) Mean age at start of ERT: 46 y Men: 56% Time since diagnosis: X REA: X Classic FD: 82% Genotype: X	ERT-naive: 100%	NA	AA 0.2 mg/kg (*n* = 248) Median follow-up: 5.2 y AB 1.0 mg/kg (*n* = 139) Median follow-up: 3.8 y	ERT-IgG	ERT-IgG; defined as ERT-IgG-positive if all measurements after Tx initiation were positive	**1.** Disease-related biomarkers (classic FD men (*n* = 92); plasma lyso-Gb3)	Retrospectively assessed during Tx (timing/frequency NR)
**Lenders 2018 [**10**]**	Retrospective (clinical records; Germany, Austria, Switzerland)	26	Mean age: 44 y Men: 100% Time since diagnosis: NR REA: NR Classic FD: 100% Genotype: 77% nonsense	ERT-naive: 100%	NA	AA: 54% AB: 46% Mean infusion dose: 70 mg Mean duration: 94 mo	NAb saturation with ERT (amount of agalsidase required to saturate NAb subtracted from infused dose at the time)	Retrospectively assessed (clinical records) for Tx period; all patients were ERT-IgG-positive; saturated patients defined as those where ERT calculated to be in excess of NAbs; non-saturated patients defined as those with NAb calculated to be in excess of ERT	**1.** Disease-related biomarkers (plasma lyso-Gb3) **2.** Renal (eGFR) **3.** CV (IVT)	Retrospectively assessed (clinical records); last clinic visit
**Mauhin, 2018 [**31**]**	Retrospective (FFABRY cohort; France)	103	ERT-IgG-positive (n = 18) Median age: 43.7 y Men: 52% Median age at diagnosis: 28.8 y Median RRA: 0.38% Classic FD: 94.4% Genotype: 44.4% missense ERT-IgG-negative (n = 27) Median age: 46.3 y Men: 100% Median age at diagnosis: 41.1 y Median RRA: 1.1% Classic FD: 44.4% Genotype: 77.8% missense	NR	NR	ERT-IgG-positive (n = 18) AA: 22% AB: 44% AA+AB: 33% Median duration: 10.6 y ERT-IgG-negative (n = 27) AA: 33% AB: 41% AA+AB: 26% Median duration: 4.3 y	ERT-IgG	Prospectively assessed at time of study inclusion Outcomes were assessed by ERT-IgG status only	**1.** Disease-related biomarkers (classic FD men; plasma lyso-Gb3) **2.** Renal (classic FD men; eGFR, kidney transplant) **3.** CV (classic FD men; hypertrophy) **5.** IRRs (classic FD men)	Retrospectively assessed (standardized online form at time of inclusion) for Tx period up to study inclusion (ERT-IgG assessment)
**Lenders 2016 [**11**]**	Retrospective (clinical records; Germany)	41	ERTi-positive (n = 18) Mean age: 44.4 y 100% men Time since diagnosis: NR Mean REA: 13.5% Classic FD: NR Genotype: 72.2% nonsense ERTi-negative (n = 23) Mean age: 41.2 y Men: 100% Time since diagnosis: NR Mean REA: 16.0% Classic FD: NR Genotype: 26.1% nonsense	NR	NR	ERTi-positive (n = 18) AA 0.2: 50% AB 1.0: 50% Mean duration: 86.2 mo ERTi-negative (n = 23) AA 0.2: 43% AB 1.0: 57% Mean duration: 58.7 mo	ERTi (NAb)	Retrospectively assessed (clinical records) for last visit; defined as ERTi-positive if mean ERTi > 50%	**1.** Disease-related biomarkers (men: plasma lyso-Gb3) **2.** Renal (various, including eGFR) **3.** CV (cardiac structure, various, including LVM)	Retrospectively assessed (clinical records) for Tx up to last visit (ERTi assessment)
**Smid 2013 [**27**]**	Retrospective (clinical records; Netherlands)	79	Mean age: 42 y Men: 52% Time since diagnosis: NR Median REA: 12.9% Classic FD: NR Genotype: 25.3% missense	NR	NR	AA 0.2: 52% AB 0.2: 15% AB 1.0: 33% Duration: NR	ERT-IgG ERTi (NAb)	Retrospectively assessed (clinical records) ERT-IgG-positive patients were positive by 3 mo of Tx; all ERT-IgG were NAbs; ERT-IgG-negative patients were negative for the whole study Outcomes were assessed by ERT-IgG status only	**5.** IRRs (including SIRRs)	Retrospectively assessed (clinical records at time of inclusion) for Tx period
**Rombach 2012 [**28**]**	Retrospective (clinical records; Netherlands)	26	ADA-positive (n = 14) Median age: 41 y Men: 100% Time since diagnosis: NR REA: NR Classic FD: 100% Genotype: NR ADA-negative (n = 12) Median age: 22.8 y Men: 100% Time since diagnosis: NR REA: NR Classic FD: 100% Genotype: NR	NR	NR	AA: 0.2 for ≥1 y AB: 0.2 for ≥1 y (part of a previous clinical trial) AB 1.0 for ≥1 y	ERTi	Retrospectively assessed serum ERTi status (performed yearly in clinic); defined as ERTi-positive if >50% neutralization of AA or AB with 5 mL plasma	**1.** Disease-related biomarkers (plasma lyso-Gb3 and Gb3 and urine Gb3)	Retrospectively assessed (clinical records); 1 y after Tx start
**Wilcox 2012 [**18**]**	Retrospective (The Fabry Registry; USA)	822	Mean age: NR Men: 70% Time since diagnosis: NR REA: NR Classic FD: NR Genotype: 55% missense (of 20 most common genotypes)	ET-naive + ERT (AB)-experienced (% split NR)	NA	AB 1.0 mg/kg	ERT-IgG	Retrospectively assessed (clinical records; timing/frequency NR); defined as ERT-IgG-negative if not detected at any time point; defined as ERT-IgG-positive (tolerized) if at least one negative test after previous positive test; defined as ERT-IgG-positive (not-tolerized) if at least one positive test at any time point and previous test was positive	**1.** Disease-related biomarkers (men; plasma Gb3) **5.** IRRs (men)	Retrospectively assessed (clinical records at time of inclusion) for Tx period
**van Breemen 2011 [**24**]**	Retrospective (clinical records; Germany)	43	Age range: 18–71 y Men: 51% Time since diagnosis: NR REA: all < 5% of normal mean Classic FD: 100% Genotype: NR	ERT-naive: 100%	NA	AA 0.2 mg/kg for ≥1 y AB 0.2 mg/kg for ≥1 y AB 1.0 mg/kg for ≥1 y	ERTi	Retrospectively assessed serum ERTi status (clinical records); baseline, and 3, 6, 9, and 12 mo after Tx	**1.** Disease-related biomarkers (plasma lyso-Gb3)	Retrospectively assessed (clinical records); baseline, and 3, 6, 9, and 12 mo after Tx start
**Benichou 2009 [**37**]**	Prospective (analysis of two trials [phase 3 and phase 4] plus their open-label extension studies; USA)	134	Phase 3 (*n* = 58) Mean age: 31 y Men: 97% Time since diagnosis: NR REA: NR Classic FD: NR Genotype: NR Phase 4 (*n* = 76) Mean age: 46 y Men: 87% Time since diagnosis: NR REA: NR Classic FD: NR Genotype: NR	NR	Phase 3 (n = 58) ADA: 97% (90% of men) Phase 4 (n = 76) ADA: 87% (76% of men)	AB 1.0 mg/kg for ≤5 y Phase 3 (*n* = 58) Median duration: 55.9 mo Phase 4 (*n* = 58) Median duration: 33.5 mo	ERT-IgG	Prospectively assessed every 2 w (phase 3 study) and 12 w (phase 4 study) during double-blind phases and every 6 mo during open-label extension phases (mean number of assessments: 24); all patients were ERT-IgG-positive; patients were grouped by peak titer during Tx (low, intermediate, high)	**1.** Disease-related biomarkers (plasma Gb3, skin Gb3) **2.** Renal (eGFR)	**1.** Prospectively assessed; plasma every 6 mo (phase 3) and 3 mo (phase 4); skin biopsies at baseline and up to 7 additional measurements from 6 to 60 mo (phase 3) **2.** Prospectively assessed; serum every 6 mo (phase 3) and 1 mo (phase 4)
**Lubanda 2009 [**32**]**	Open-label, single-arm trial (EU; multicenter)	21	Mean age: 34.0 y Men: 100% Median age at diagnosis: 28 y REA: NR Classic FD: NR Genotype: 52% missense	NR	NR	AB 1.0 mg/kg for 6 mo then AB 0.3 mg/kg for 18 mo	ERT-IgG	Prospectively assessed at baseline and every 6 w during Tx; defined as ERT-IgG-negative if no positive test at any assessment; ERT-IgG-positive patients divided into peak titer groups (lowest, intermediate, highest)	**1.** Disease-related biomarkers (urine Gb3)	Prospectively assessed; every 6 w during Tx
**Ohashi 2007 [**30**]**	Study design NR (Japan)	14	ERT-IgG-positive (n = 7) Median age: 37 y Men: % NR Time since diagnosis: NR REA: NR Classic FD: 100% Genotype: NR ERT-IgG-negative (n = 7) Median age: 26 y Men: % NR Time since diagnosis: NR REA: NR Classic FD: 100% Genotype: NR	NR	NR	AB 1.0 mg/kg for ≤30 mo	ERT-IgG	ERT-IgG status assessed at baseline	**1.** Disease-related biomarkers (urine Gb3) **2.** Renal (eGFR, proteinuria, serum creatinine)	**1.** Baseline and every 3 mo (up to 30 mo) **2.** Baseline
**Vedder 2008 [**21**]**	Study design NR (Netherlands, Germany)	59	AA 0.2 mg/kg (*n* = 18) Mean age: 47 y Men: 56% Time since diagnosis: NR REA: NR Classic FD: NR AB 0.2 mg/kg (*n* = 13) Mean age: 49 y Men: 62% Time since diagnosis: NR REA: NR Classic FD: NR AB mg/kg 1.0 (*n* = 21) Mean age: 48 y Men: 48% Time since diagnosis: NR REA: NR Classic FD: NR Males (non-switch study; *n* = 28) REA: 0–6.4 Genotype: 53% missense Males (switch study; *n* = 7) NR	Patients in switch study (*n* = 7) previously received AA (*n* = 3) or AB (*n* = 4) 0.2 mg/kg	NR	AA 0.2 mg/kg for ≥1 y (*n* = 18) AB 0.2 mg/kg for ≥1 y (*n* = 13) AB 1.0 mg/kg for ≥12 mo (*n* = 21) AB 1.0 mg/kg for ≥1 y (switch study; *n* = 7)	ERT-IgG ERTi (NAb)	Defined as ERT-IgG-positive if detected after 6 mo; NAb (>50% reduction in ERT activity with 1 µL of serum) present in 17/18 men (non-switch study) who were ERT-IgG-positive Outcomes were assessed by ERT-IgG status only	**1.** Disease-related biomarkers (plasma Gb3, urine Gb3) **2.** Renal (eGFR) **3.** CV (LVM)	Baseline and after 1 y of Tx
**Vedder 2007 [**20**]**	Open-label RCT (Netherlands)	34	Median age: 44 AA (*n* = 18) Median age: NR Men: 50% Time since diagnosis: NR REA: NR Classic FD: NR Genotype: NR AB (*n* = 16) Mean age: NR Men: 56% Time since diagnosis: NR REA: NR Classic FD: NR Genotype: NR	NR	AA (n = 18) ADA: 22% AB (*n* = 16) ADA: 38%	AA 0.2 mg/kg for ≥1 y	ERT-IgG	Prospectively assessed; baseline	**1.** Disease-related biomarkers (men; plasma and urine Gb3)	Prospectively assessed at baseline, and at 1 y and 2 y
**Linthorst 2004 [**13**]**	Retrospective (clinical records; Netherlands)	18	Mean age: NR Men: 89% Time since diagnosis: NR REA: 0–16.1 mmol/mg/h Classic FD: NR Genotype: 33% missense	NR	NR	AA 0.2 mg/kg for 6–12 mo AB 0.2 mg/kg for 6–12 mo AB 1.0 mg/kg for 6–12 mo	ERT-IgG ERTi (NAb)	Retrospectively assessed (clinical records) at pre-Tx and 6 mo after Tx (and 12 mo after Tx in some patients); all ERT-IgG-positive patients were NAb-positive Outcomes were assessed by ERT-IgG status only	**1.** Disease-related biomarkers (urine Gb3) **5.** IRRs	**1.** Retrospectively assessed (clinical records at time of inclusion); baseline, and 6 mo (*n* = 11) and 12 mo (*n* = 6) during Tx **5.** Retrospectively assessed (clinical records at time of inclusion) for Tx period (*n* = 18); frequency NR

AA, agalsidase alfa; AB, agalsidase beta; ADA, antidrug antibody; ADR, adverse drug reaction; AE, adverse event; AF, animal component-free; CFDI; Canadian Fabry Disease Initiative; CV, cardiovascular; eGFR, estimated glomerular filtration rate; ERT, enzyme replacement therapy; ERTi, ERT inhibition; FD, Fabry disease; Gb3, globotriaosylceramide; HR, hypersensitivity reaction; IgG, immunoglobulin G; IRR, infusion-related reaction; LLN, lower limit of normal; LVM, left ventricular mass; lyso-Gb3, globotriaosylsphingosine; mo, months; NAb, neutralizing antibody; NR, not reported; PMS, post-marketing authorization study; RB, ‘roller-bottle’ formulation (contained animal proteins); RCT, randomized controlled trial; REA, residual enzyme activity; RRA, relative residual activity ([activity with patient serum]/[activity with control] × 100%); SADR, serious adverse drug reaction; SAE, serious adverse event; SD, standard deviation; SHR, severe hypersensitivity reaction; SIRR, serious infusion-related reaction; Tx, treatment; y, years

For the purposes of this review, ERTi refers to ERT inhibition (via serum-mediated ERTi assays; used to determine the presence of neutralizing antibodies [NAbs]), ERT-IgG refers to antibodies that target the ERT backbone and not other moieties (regardless of NAb status), and ADA refers to ADAs, regardless of whether they are ERTi or ERT-IgG. Many studies included assessments of ERTi in addition to assessing ERT-IgG; however, most (with some notable exceptions [10, 11, 22–24, 28]) based their analyses of outcomes by ADA status on the presence or absence of ERT-IgG only. Of note, ADA assessments, assessment protocols, and ADA positivity definitions varied between the included studies; clear reporting of ADA assessment protocols (including assessment timings) was infrequent. For example, some studies defined ADA positivity based on ADA parameters at baseline; other studies based their definitions on ADA positivity parameters met at any time during the study.

Overall, eight studies reported ADA status at baseline [9, 19–21, 33, 35–37]. Patient populations in these studies were predominantly adult (cross-study mean age range: 31.0–48.9 years) and more commonly male (cross-data set median: 61% male). Fabry disease phenotype was not reported for any of the study populations, and none of these studies explicitly reported on rates and progression of seroconversion and reversion back to ADA-negative status, although several highlighted the male bias in ADA development and the change in titers with time. This review pools results for ADA-positive subgroups, agnostic of how ADA positivity was defined in the respective studies; however, where contributing studies defined ADA status based on presence/absence of ADAs at baseline, it is noted.

### Fabry disease-related biomarkers by ADA status

**Fig. 2:**
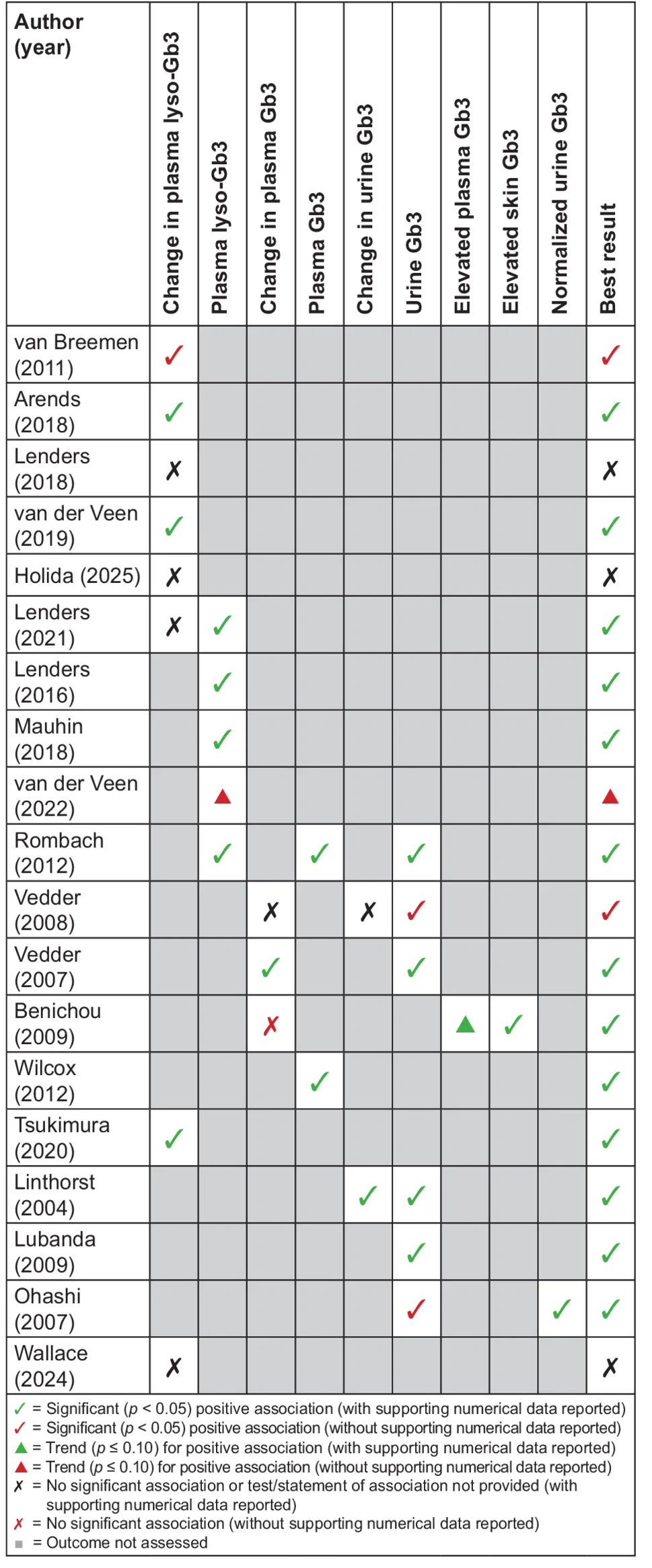
Summary panel of disease-related biomarker outcomes with ERT by ADA status. ADA, antidrug antibody; ERT, enzyme replacement therapy; Gb3, globotriaosylceramide; lyso-Gb3, globotriaosylsphingosine

Of the included studies, 19 reported on Fabry disease-related biomarkers stratified by ADA status [10, 11, 13, 18–24, 26, 28, 30–32, 34, 36–38], of which 17 reported at least one statistically significant positive association between ADA positivity and higher disease-related biomarker levels (Fig. 2).

The most commonly evaluated disease-related biomarker was plasma lyso-Gb3, which was assessed in 12 out of 19 studies [10, 11, 19, 22–24, 26, 28, 31, 34, 36, 38]. The proportion of ADA-positive patients at baseline was reported for four of these studies. Three of these studies involved populations who had received prior ERT (‘ERT-experienced’); the proportions of ADA-positive patients at baseline in the studies were 34%, 33% and 59%, respectively [19, 28, 34]. The fourth study (a pooled open-label extension of a phase 3 [*n* = 58] and a phase 4 [*n* = 76] trial) included both ERT-experienced patients and patients who had received no prior ERT (‘ERT-naive’) [22]. Overall, 97% of patients from the phase 3 trial and 87% from the phase 4 trial were ADA-positive at study baseline, baseline ADA status was not reported by prior ERT exposure status [22]. Eight of the 12 studies indirectly or directly compared the change from baseline in plasma lyso-Gb3 levels between ADA-positive and ADA-negative patients [9, 10, 19, 22, 24, 26, 36, 38]. Of these studies, five demonstrated statistically significant associations between ADA positivity (or high inhibition titers) and increased plasma lyso-Gb3 levels [26, 34], or between ADA positivity and a smaller reduction in plasma lyso-Gb3 levels over time [22, 24, 38]. In three of these, statistically significant associations were observed for a subpopulation only: in one study, significant association between ADA positivity and increased plasma lyso-Gb3 levels was described for patients who switched from agalsidase alfa or beta to pegunigalsidase alfa, but not for those who received agalsidase beta throughout [34]; in another, ADA positivity was significantly associated with a smaller reduction in plasma lyso-Gb3 levels over time for patients treated with 0.2 mg/kg agalsidase alfa or beta, but not for those treated with 1.0 mg/kg agalsidase beta [24]; the remaining study found significant association with both of these variables in patients treated with agalsidase alfa but not in those treated with agalsidase beta [38]. Two out of eight studies did not directly compare the lyso-Gb3 levels in patients with or without ADA saturation (‘saturated’ patients were those who received a sufficient dose of agalsidase alfa or beta to bind and neutralize available ADAs) [10, 19]. In one of these studies, association was suggested between ADA saturation and greater reductions in lyso-Gb3 levels [10], while the other study reported that notable lyso-Gb3 increases from baseline (exceeding 20% and 10 nM) were likely to occur in patients with a baseline urine protein-to-creatinine ratio of ≥1 g/g and ADA-positive status [19]. Finally, one study found a numerical difference in the plasma lyso-Gb3 increase between ADA-positive and ADA-negative patients, but no significance test was reported [36]. In this study, patients were switched from agalsidase alfa (0.2 mg/kg every 2 weeks [E2W]) or beta (1.0 mg/kg E2W) to pegunigalsidase alfa (2 mg/kg every 4 weeks for 52 weeks), numerical data were reported favoring a smaller increase in plasma lyso-Gb3 in ADA-negative patients (median 2.7 nM) than in ADA-positive patients (median 6.3 nM) [36]. Six of the 12 studies compared plasma lyso-Gb3 levels after ERT between ADA-positive and ADA-negative subgroups [11, 23, 28, 31, 34]; five studies reported significantly higher lyso-Gb3 levels in ADA-positive patients than in ADA-negative patients, and one study reported a positive but nonsignificant association (*p* = 0.06) [23]. One of these studies reported a significant association only for patients who switched from reduced dose of agalsidase beta to agalsidase alfa, while those treated with agalsidase beta throughout had comparable lyso-Gb3 levels regardless of their ADA status [9]).

Plasma Gb3 levels were assessed in relation to ADA status in five out of the 19 studies [18, 20, 21, 28, 37]. The proportion of ADA-positive patients at baseline was reported for four of these studies: two included ERT-experienced patients only and two did not report on previous ERT exposure. Among the ERT-experienced study populations, 35% and 59% were ADA-positive at baseline [21, 28]; in the studies without prior treatment data reported, 34% and 90% of patients were ADA-positive at baseline [20, 37]. Three of the five studies reporting on plasma Gb3 levels compared the change in level from baseline for ADA-positive and ADA-negative patients [20, 21, 37]; one reported a significantly lower reduction in Gb3 in ADA-positive (vs ADA-negative) patients [20] and two reported no association [21, 37]. One of the studies reported no statistically significant association between ADA status and plasma Gb3 levels, but a statistically significant association between anti α-Gal A IgG titers and Gb3 deposition in the superficial dermal capillary endothelial cells during treatment [37]. Two of the five studies compared plasma Gb3 levels after ERT between ADA-positive and ADA-negative patients [18, 28]; both reported significantly higher plasma Gb3 levels in ADA-positive patients (one study reported these findings in male but not in female participants [18]).

Urine Gb3 levels were assessed in relation to ADA status in six studies [13, 20, 21, 28, 30, 32]. The proportion of ADA-positive patients at baseline was reported for three studies: two included ERT-experienced-patients only (38% and 59% of patients were ADA-positive at baseline) [21, 28]; one did not report previous treatment exposure (35% of patients were ADA-positive at baseline) [20]. Of the six studies comparing urine Gb3 levels after ERT of ADA-positive and ADA-negative patients, all reported significantly or relatively higher levels in ADA-positive patients [13, 20, 21, 28, 30, 32]. One of these studies also reported a significantly lower rate of urine Gb3 normalization (defined as urinary Gb-3 reduced below 1.3 µg/mg creatinine on two consecutive occasions) in ADA-positive versus ADA-negative patients [30]. Two of the six studies reported change in baseline urine Gb3 levels for ADA-positive and ADA-negative patients [13, 21]. In both studies urine Gb3 levels increased from baseline in the ADA-positive subgroup and decreased in the ADA-negative group (in patients treated with 0.2 mg/kg agalsidase alfa/beta, but not those treated with 1.0 mg/kg agalsidase beta [21]). In one study, this divergence resulted in a statistically significant between-group difference at 6 months, which had not been present at baseline [13]. The second study reported only numerical changes in urine Gb3 levels; and did not provide statistical comparisons between the ADA subgroups [21].

### Renal outcomes by ADA status

**Fig. 3:**
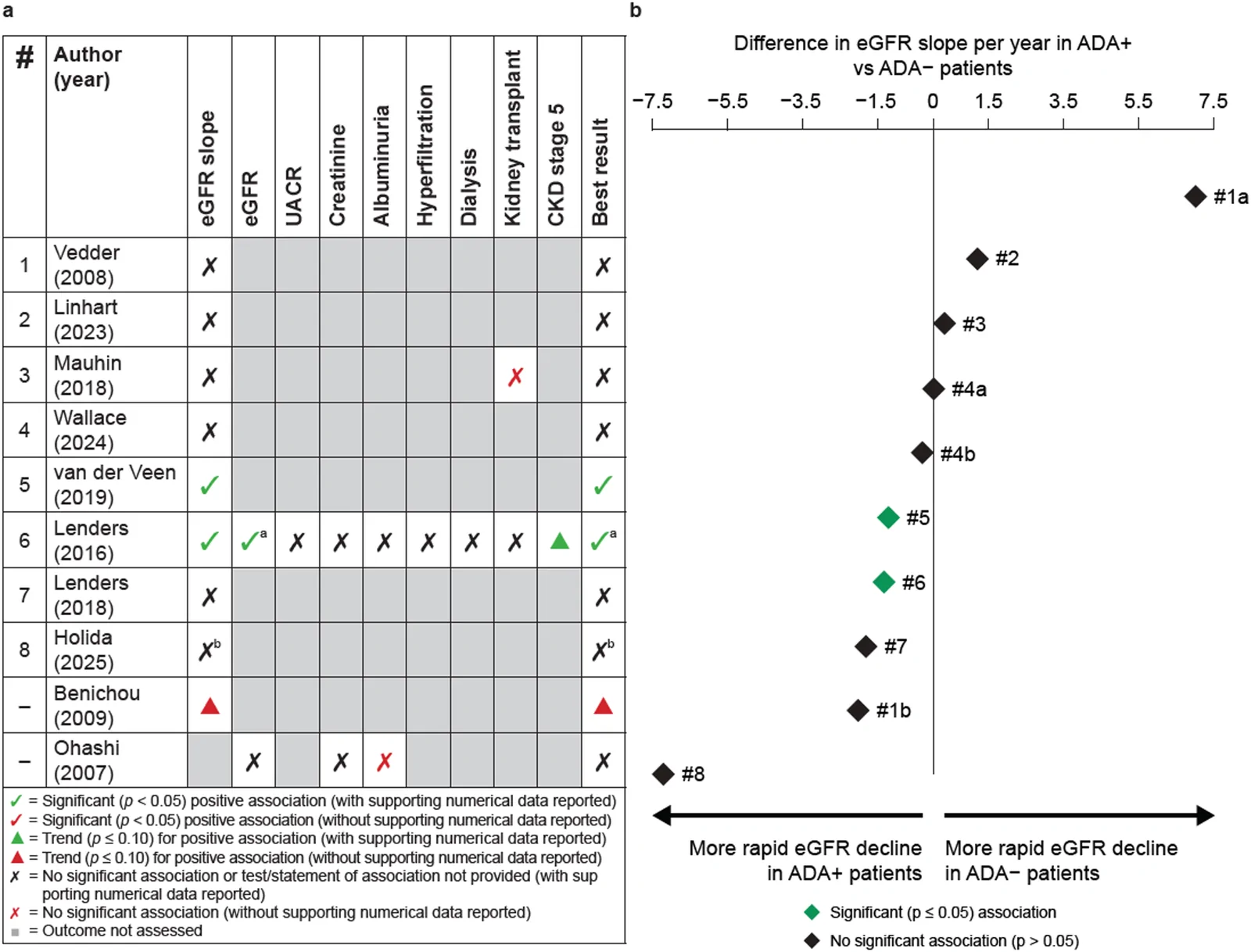
Summary of renal outcomes with ERT by ADA status (**a**) and difference in eGFR slope per year between ADA-positive and ADA-negative patients in the subset of studies (#1–8) reporting comparable data (**b**). ^a^Mixed-model analysis adjusted for age, ERT duration, nonsense mutations, and use of renin–angiotensin–aldosterone system blockers and diuretics. ^b^Numerical difference suggesting a potential positive association between rate of eGFR decline and ADA positivity but no statistical test conducted. Data from two treatment groups are reported in b for Vedder et al. 2008: AB 1.0 mg/kg (#1a) and AA or AB 0.2 mg/kg (#1b). Data from two treatment groups are reported in b for Wallace et al. [19]: AB 1.0 mg/kg (#4a) and PA 1.0 mg/kg (#4b). AA, agalsidase alfa; AB, agalsidase beta; ADA, antidrug antibody; CKD, chronic kidney disease; eGFR, estimated glomerular filtration rate; ERT, enzyme replacement therapy; PA, pegunigalsidase alfa; UACR, urinary albumin-to-creatinine ratio

Ten studies evaluated renal outcomes stratified by patient ADA status (Fig. 3a) [10, 11, 19, 21, 22, 30, 31, 33, 36, 37]. Four of these studies reported the proportion of ADA-positive patients at baseline: across three studies (of ERT-experienced populations), the proportion of patients who were ADA-positive at baseline ranged from 9% to 46% [19, 21, 33]; in the fourth study, which included both ERT-experienced and ERT-naive patients, 59% of patients were ADA-positive at baseline [22].

Nine of the 10 identified studies reported data on the estimated glomerular filtration rate (eGFR) slope per year (Fig. 3a) [10, 11, 19, 21, 31, 33, 36, 37]. Two of the nine studies reported a significant association between neutralizing ADAs (ERTi) and more rapid eGFR decline [10, 11], and one reported a trend toward more rapid eGFR decline with increasing ADA (ERT-IgG) titer (*p* = 0.10) [37]; however, six studies found either no association (2 studies) [21, 31] or reported data without a statistical analysis (4 studies all ERT-IgG) [10, 19, 33, 36].

One of the two studies that reported a significant association between neutralizing ADAs (ERTi) and more rapid eGFR decline also reported significantly lower eGFR in ERTi-positive versus ERTi-negative patients at last visit (64.1 vs 93.3 mL/min/1.73 m^2^; *p* = 0.04), based on a mixed-model analysis that adjusted for age, ERT duration, nonsense mutations, and use of renin–angiotensin–aldosterone system blockers and diuretics [11]. This same study also reported a trend toward a higher incidence of chronic kidney disease stage 5 in ERTi-positive versus ERTi-negative patients (16.7% [3/18] vs 0% [0/23]; *p* = 0.08); no significant effects on urine albumin-to-creatinine ratios, creatinine levels, or proportions of patients with albuminuria, hyperfiltration, or undergoing dialysis/kidney transplantation were noted (Fig. 3a).

Eight of the 10 studies that evaluated associations between ADA status and eGFR slope provided supporting numerical data [10, 11, 19, 21, 22, 31, 33, 36]; two reported data separately for comparator treatment groups, resulting in 10 data sets overall (Fig. 3b). Three of the eight data sets indicated a more rapid numerical decline in eGFR in ADA-positive patients (ERTi-positive [11, 22] or ERT-IgG [36]) than in ADA-negative patients, two indicated no difference (both based on ERT-IgG) [19, 31], and three indicated a slower numerical (but statistically nonsignificant) decline in eGFR in ADA-positive or ADA-saturated patients (two based on ERT-IgG) [21, 33], one based on saturated vs unsaturated ADA sites following agalsidase infusion [10]). One of the two studies that reported a slower numerical decline in eGFR in ADA-positive patients was a switch study in which patients who had received agalsidase alfa 0.2 mg/kg treatment for 2 years switched to pegunigalsidase alfa 1 mg/kg for 12 months [33]; the other was a comparative study of higher-dose agalsidase beta (1.0 mg/kg) versus lower-dose agalsidase alfa or beta (0.2 mg/kg) [21]. The switch study was unique in that it reported a substantial increase in eGFR over 12 months with pegunigalsidase alfa in both ADA-positive and ADA-negative patients [33]. However, interpretation of this finding is limited given the use of a higher-dose ERT post-switch and a methodology for eGFR assessment post-switch that differed from that used pre-switch [33]. Although not directly comparable, other studies have shown declining eGFR over 12 months regardless of ADA status. The comparative study in the present data set that reported a slower eGFR decline in ADA-positive (vs ADA-negative) patients only did so for the higher-dose treatment arm (agalsidase beta 1.0 mg/kg vs agalsidase alfa/beta 0.2 mg/kg); the 1.0 mg/kg treatment arm also showed a significant decrease in plasma Gb3 in ADA-positive patients but not in ADA-negative patients [21].

### Cardiovascular outcomes by ADA status

**Fig. 4:**
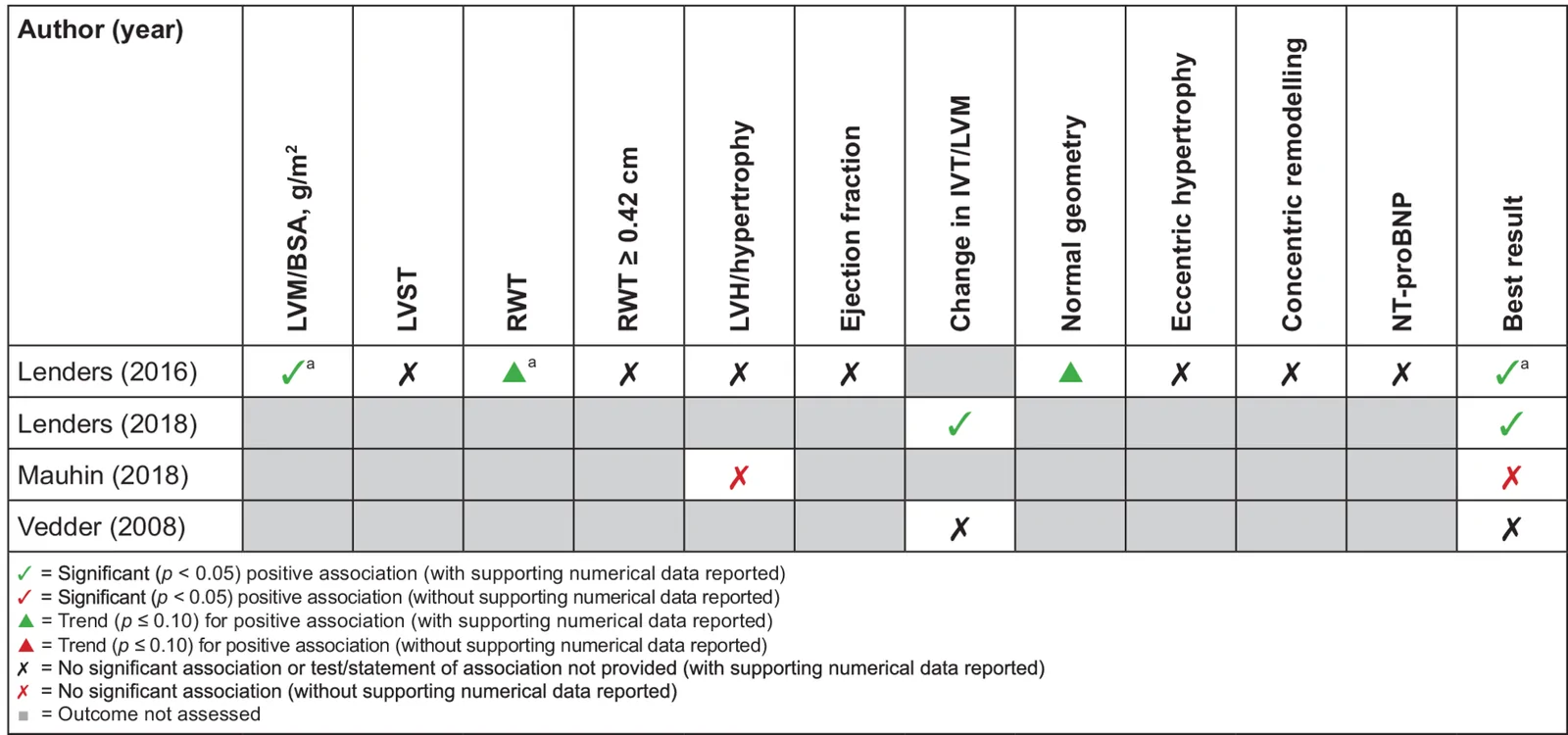
Summary panel of cardiovascular outcomes with ERT by ADA status. ^a^Mixed-model analysis adjusted for age, ERT duration, use of renin–angiotensin–aldosterone system blockers, nonsense mutations, body weight, and systolic and diastolic blood pressure. ADA, antidrug antibody; BSA, body surface area; ERT, enzyme replacement therapy; IVT, intraventricular wall thickness; LVH, left ventricular hypertrophy; LVM, left ventricular mass; LVST, left ventricular septum thickness; NT-proBNP, N-terminal pro-brain natriuretic peptide; RWT, relative wall thickness

In total, four identified studies included data on cardiovascular-related outcomes by patient ADA status [10, 11, 21, 31]. One study reported the proportion of ADA-positive patients at baseline and included ERT-experienced patients only (the proportion of ADA-positive patients ranged from 22% to 46% across treatment groups) [21]. Two of the four studies reported a significant positive association between ADA positivity and cardiovascular-related outcomes (left ventricular mass [LVM] indexed to body surface area [11] and change in interventricular septum thickness [10]) and two reported no significant association with the cardiovascular-related outcome evaluated (cardiac hypertrophy and stroke [31] and change in LVM [21]) (Fig. 4).

The study that reported a significant positive association between ADA positivity and increased LVM (105.8 [ERTi-positive] vs 71.5 [ERTi-negative]; *p* = 0.02) used a mixed-model analysis that adjusted for age, ERT duration, use of angiotensin–aldosterone system blockers, nonsense mutations, body weight, and systolic and diastolic blood pressure [11]. This study also reported trends toward increased relative wall thickness (0.57 vs 0.42 cm; *p* = 0.07 [same mixed-model adjustment as for LVM]) and less frequent normal cardiac geometry (35.7% vs 68.2%; *p* = 0.09; unadjusted) in ERTi-positive versus ERTi-negative patients, respectively [11].

### IRR and safety outcomes by ADA status

**Fig. 5:**
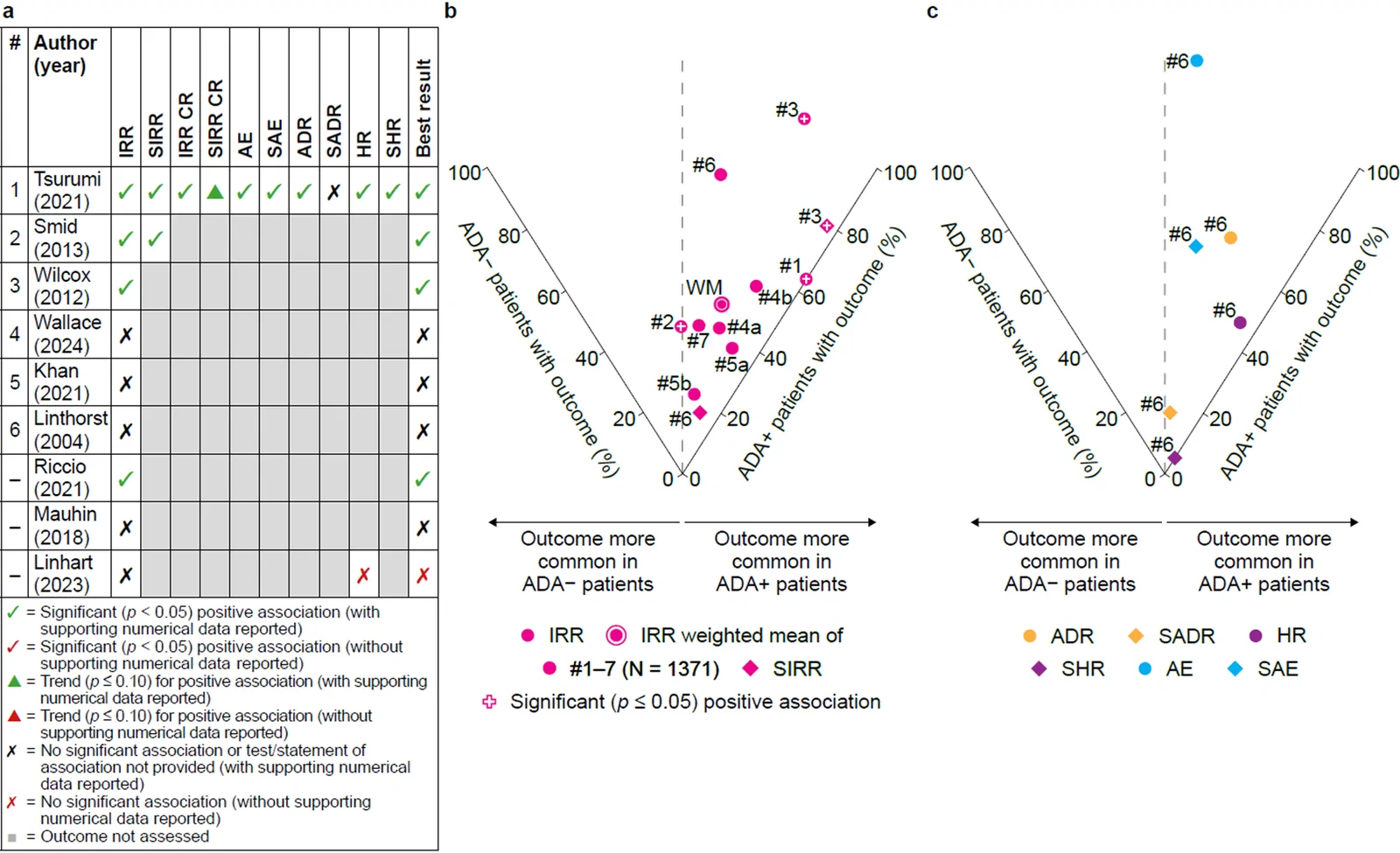
Summary of IRR/AE outcomes with ERT by ADA status (**a**) and differences in proportions of patients with IRR/AE outcomes between ADA-positive and ADA-negative patients in the subset of studies (#1-7) reporting comparable data (**b**, **c**). Data points in left-hand triangles = outcome more frequent in patients who are ADA−. Data points in the right-hand triangles = outcome more frequent in patients who are ADA+. Corresponding study (#): 1. Linthorst et al. [13] [men]. 2. Khan et al. [35]. 3. Smid et al. [27]. 4. Wallace et al. [19] [4a. PA, men; 4b. AB, men]. 5. Wilcox et al. [18] [5a. men; 5b. women]. 6. Tsurumi et al. [25]. 7. Linhart et al. [33]. AB, agalsidase beta; ADA, antidrug antibody; ADA−, ADA-negative; ADA+, ADA-positive; ADR, adverse drug reaction; AE, adverse event; CR, causal relationship; ERT, enzyme replacement therapy; IRR, infusion-related reaction; HR, hypersensitivity reaction; PA, pegunigalsidase alfa; SADR, serious adverse drug reaction; SAE, serious adverse event; SHR, severe hypersensitivity reaction; SIRR, serious infusion-related reaction; WM, weighted mean

Nine studies assessed IRRs and/or AEs by ADA (ERT-IgG) status [13, 18, 19, 25, 27, 29, 31, 33, 35]. With the exception of one RCT [19], eight studies had non-randomized designs; no studies randomized patients by baseline ADA status. Four studies included ERT-experienced populations [13, 19, 27, 33], and five studies included both ERT-experienced and ERT-naive patients [18, 25, 29, 31, 35]. The proportion of ADA-positive patients at baseline was reported for four studies: three included ERT-experienced patients only (the proportion of ADA-positive patients ranged from 7% to 34%) [19, 27, 35], and the fourth study included both ERT-experienced and ERT-naive patients (9% of patients were ADA-positive at baseline) [33]. Across the included studies, the median proportion of patients who experienced IRRs was 24% (cross-study range: 11–57%). Several studies suggested a possible association between ADA status and IRR occurrence (Fig. 5a). The weighted mean approach was used descriptively to reduce the influence of small studies’ contributions so they are not equal to those of larger studies. These values are presented for descriptive purposes only and should not be interpreted as pooled effect estimates.

Four of the nine studies reported statistically significant positive associations between IRR occurrence and ERT-IgG, of which one study included only ERT-experienced patients and three studies included both ERT-experienced and ERT-naive patients [18, 25, 27, 29]. Two of these studies also assessed serious IRRs and reported positive associations between ERT-IgG status and serious IRR occurrence [25, 27].

Seven studies stratified the proportions of patients with IRRs by ERT-IgG status (Fig. 5b). Five reported higher rates of IRRs in ERT-IgG-positive patients than in ERT-IgG-negative patients (three studies included ERT-experienced populations only [22, 24, 38]; two studies included mixed ERT-naive and ERT-experienced populations [18, 25]; one study [18] reported significant positive associations in male but not in female participants). Two studies showed similar rates of IRRs between these groups (one of these studies included only ERT-experienced patients; the other included a mixed ERT-naive and ERT-experienced population) [33, 35]. Another study did not include data on proportions of patients with IRRs by ADA status, but did report a numerically higher (but nonsignificant) probability of IRR occurrence in ADA-positive (vs ADA-negative) patients with classic Fabry disease (odds ratio of 4.2; *p* = 0.32) [31]. Across all studies, the sample size weighted mean proportion of patients with IRRs was 38% (cross-study range: 24–89%) in the pooled ADA-positive population (*n* = 647), and 18% (cross-study range: 0–26%) in the pooled ADA-negative population (*n* = 724). One study that reported a higher rate of IRRs in ERT-IgG-positive versus ERT-IgG-negative patients also reported higher incidences of IRRs in ADA-positive versus ADA-negative patients (pegunigalsidase alfa: 0.9 vs 0.3 events/100 infusions, respectively; agalsidase beta: 7.5 vs 2.2 events/100 infusions, respectively) [19].

Other safety outcomes were assessed in two studies (Fig. 5) [25, 33]. Both studies assessed hypersensitivity reactions (HRs): one reported significantly more HRs (42 vs 5 patients, respectively; *p* < 0.001) and more severe HRs (5 vs 0 patients, respectively; *p* = 0.025) in ERT-IgG-positive patients than in ERT-IgG-negative patients [25]; the other reported no significant association between ERT-IgG status and incidence of HRs or severe HRs [33]. The latter study did, however, note that two patients who had a severe HR were IgE-positive, consistent with the known role of this antibody type in anaphylactic reactions [33]. One of the studies assessed AEs, serious AEs, adverse drug reactions (ADRs), and serious ADRs; all of these outcomes (with the exception of serious ADRs) were significantly positively associated with ERT-IgG (Fig. 5a) and all were more common in ERT-IgG-positive patients than in ERT-IgG-negative patients (AEs: 72 vs 55 patients; serious AEs: 43 vs 27; ADRs: 53 vs 20; serious ADRs: 11 vs 8 patients, respectively) (Fig. 5c) [25].

## Discussion

To our knowledge, this is the first systematic evaluation of the relationship between ADAs and specific clinical and subclinical outcomes in patients with Fabry disease receiving ERT. Across most of the included studies, ADA positivity was reportedly associated with higher levels of Fabry disease-related biomarkers, potentially indicative of diminished ERT activity, and signals for reduced ERT efficacy and safety. Although the observed associations were broadly consistent within the eligible literature, the results were heterogeneous and do not demonstrate causality due to the potential effect of confounding variables. Additionally, the included studies were observational and not designed a priori to investigate temporal relationships between ADA development and outcomes of interest. Together, the study design and the heterogeneity of findings represent limitations that prevented assessment of causality in this review. However, they highlight an important area for future research: prospective and mechanistic studies to define the clinical implications of ADA development.

Despite these limitations, the review findings challenge the results from earlier publications, which reported a lack of clinical impact of ADAs [19, 21, 33]. The difference in findings may reflect the heterogeneous study designs, assessments, and patient populations. For instance, many studies included in this review did not measure NAbs or record the type of Fabry disease in the patients. Considering that renal and biomarker outcomes are more directly affected by NAbs, and that patients with classic Fabry disease are both more likely to have ADAs and more likely to have worse clinical outcomes than those with other types of Fabry disease, this may have contributed to the lack of clinical impact of ADAs reported in prior publications. The heterogeneity of both study designs and findings highlights the need for future studies that robustly analyze the relationship between ADAs and clinical outcomes of people living with Fabry disease.

In the context of the Fabry disease-related biomarkers evaluated, the consistently higher levels in ADA-positive patients likely reflect the highly proximal nature of the biomarkers assessed (Gb3 and lyso-Gb3) to ERT activity, thereby allowing signals of potential association to be detected despite substantial background noise due to study heterogeneity. This review provides a useful consolidation of the available evidence to support further investigations of the relationship.

The efficacy analysis was more equivocal. The majority of studies reporting on renal outcomes found no statistically significant association between evaluated outcomes and ADA status. This is perhaps not surprising, given that renal (and cardiovascular) outcomes are more varied in nature and in their method of assessment. Additionally, their likely association with long-term ERT exposure is harder to evaluate in a short-term study, particularly given that the association between ADA status and renal outcomes was not an a priori objective of any of the included studies. Nevertheless, numerical or statistically significant evidence of more rapid eGFR decline in ADA-positive versus ADA-negative patients was observed in more than half of the ten data sets included. Among the studies that, conversely, reported evidence of a positive association between ADA negativity and higher rate of eGFR decline, two reported data suggesting that treatment dose may have contributed to the observed associations. In one of these studies, which reported results by treatment arm (agalsidase beta [1.0 mg/kg] and agalsidase alfa/agalsidase beta [0.2 mg/kg]), the numerical association between increased eGFR decline in ADA-positive versus ADA-negative patients was only observed in the agalsidase beta 1.0 mg/kg data set. This also showed a significant decrease in plasma Gb3 in ADA-positive patients but not ADA-negative patients, against expectations and in contrast to other studies [21]. The second study was a ‘switch’ study in which patients receiving agalsidase alfa 0.2 mg/kg treatment were switched to pegunigalsidase alfa 1 mg/kg; the eGFR slope increased in both ADA-positive and ADA-negative patients (eGFR slopes decreased in both groups in all of the other studies) [33]. Although, the effect of differing pre- and post-study start eGFR methodology needs to be kept in mind, one possible explanation is that ADA-positive patients may have had greater capacity for improvement following the switch to a higher-dose and potentially more efficacious enzyme. The observed change in eGFR slope could also reflect the effect of dose escalation (from 0.2 mg/kg to 1.0 mg/kg), indicating that higher ERT doses may overcome some of the negative impact of ADAs on treatment efficacy. Alternatively, the lower-dose regimen (0.2 mg/kg) may have been insufficient to produce meaningful differences between ADA subgroups [33]. However, comparing eGFR slopes between studies with different designs (i.e. switching treatment vs continuous treatment) requires caution due to possible differences in eGFR measurements before and after switch. Overall, the lack of a strong association between ADA status and renal outcomes may reflect the inclusion of non-neutralizing IgG-positive patients in the ADA-positive group; with limited studies reporting the NAb status of patients, a separation of results by ADA subtype was not possible. We recommend that future studies assess renal outcomes in patients with neutralizing and non-neutralizing ADAs separately. It is also potentially noteworthy that three [10, 11, 22] of the six data sets [10, 11, 19, 21, 22, 36] that showed a trend (*p* ≤ 0.10) toward accelerated eGFR decline in ADA-positive patients were also the only studies that defined ADA status using ERTi (rather than ERT-IgG); two of these three studies were the only ones to report statistical significance (*p* ≤ 0.05) [11, 22]. All three of these studies had aspects to their design that aimed to minimize the potential for confounding due to more severe disease in patients with ADAs (an issue for all studies in this review, including the RCTs because of the lack of randomization by ADA status). One study included only patients with classic Fabry disease [22]; one included only patients who were ADA-positive and compared outcomes between patients who were ADA-saturated with those who were not [10]; and the third used a mixed-effects model to adjust for confounding (the only one of any of the identified studies to do so) [11]. Of note, the latter two studies were also the only ones (out of four reporting data) to show a significant positive association between ADA positivity and poorer cardiovascular outcomes [10, 11].

All studies that provided a test and/or statement of association for the relationship between ADA positivity and IRR risk reported a significant positive association [18, 25, 27, 29]. The proportion of patients affected by IRRs in the pooled ADA-positive population was more than twice that in the pooled ADA-negative population (38% vs 18%, respectively). In addition, direct examination of the numerical data within the literature found that proportions of patients experiencing IRRs were consistently appearing to be higher in ADA-positive versus ADA-negative patient subgroups. However, it is noteworthy that IRRs still occurred in ADA-negative patients, suggesting that not all IRRs are necessarily related to the effects of ADAs. It is also relevant to note that ADA assessment in the studies was limited to IgG-class antibodies; therefore, some IRRs may have been triggered by undetected IgM-, IgE-, or NAb-class ADAs. The fact that the association between IRR and ADA positivity was evident despite most studies relying on detection of ERT-IgG rather than ERTi may reflect that non-neutralizing antibodies can still promote the formation of immune complexes and, thus, inflammatory responses, whereas effects on renal and cardiovascular outcomes may be more dependent on the ability of antibodies to inhibit ERT activity. Similar to Fabry disease-related biomarkers of ERT activity, IRR events are also (by definition) highly proximal to infusions, which may strengthen the ability to detect associations.

An additional consideration when interpreting results of this review is the heterogeneity of ADA definitions and assay methodologies used across studies. For example, most studies classified patients as ‘ADA-positive’ based on IgG results, while only a few reported other classes of ADAs, such as neutralizing antibodies. It is possible that associations between ADA status and IRR risk were detected because of the direct link between IRRs and the total IgG responses. By contrast, renal and biomarker outcomes are more directly linked to neutralizing antibodies, so strength of associations may have been reduced by the inclusion of patients with non-neutralizing IgG in the ADA-positive category in some analyses. Stratification of results by ADA definition and assay type in this review was not possible due to the inconsistent and heterogeneous study designs and reporting of results. We suggest that future studies incorporate standardized ADA characterization, especially explicit differentiation of neutralizing versus non-neutralizing antibodies.

Although the lack of standardized ADA definitions and assay methodologies may affect the interpretation of the results, this variability itself illustrates the need for a more uniform approach to ADA reporting in future studies to fully understand the associations between ADAs and clinical outcomes of people living with Fabry disease.

Additionally, ADA development is heavily dependent on a patient’s sex, genotype (e.g. their cross-reactive immunological material status or the presence of nonsense mutations), as well as their disease phenotype and severity. Although variability in the reporting of genotypes, disease classification, and severity across studies introduces some limitations in cross-study comparisons, the available evidence still provides important insights into patterns of ADA incidence and associated outcomes. Notably, in several large studies in which genotype and/or classic Fabry disease classification were not reported, the ability to stratify ADA risk according to mutation type is limited. This is particularly relevant given the established relationship between genotype (e.g. cross-reactive immunological material [CRIM] status and nonsense mutations) and immunogenicity. As such, the absence of these data constrains more granular interpretation of ADA risk across patient subgroups. At the same time, this variability highlights the need for more standardized reporting to better distinguish whether observed associations are driven by the immunogenicity of specific mutations, differences in the natural history of Fabry disease subtypes, or true ADA-mediated effects.

The aim of this SLR was to evaluate associations between ADA status and specific outcomes, and to identify remaining evidence needs. Key elements of PRISMA-standard SLR were undertaken, including systematic searches and screening, quality assessment, including at least one person for data extraction and another for data checks. The literature search was restricted to English language articles; however only 1/1763 articles (0.06%) was excluded owing to language and this exclusion criterion did not limit the geographic scope of the research. Interpretation of the findings of this review is limited by factors such as the small sample sizes involved in the studies, the retrospective and *post hoc* design of many of the analyses and their primary focus on only IgG-class ADAs, and differences in study design (including population eligibility [e.g. prior ERT exposure] and outcomes analyzed), all of which limited the ability to draw cross-study conclusions. One of the key challenges in interpreting the data was the inconsistent use (and/or incomplete reporting) of standardized ADA assessment protocols (assays, timings, seroconversion/seroreversion) across the studies, preventing evaluation of temporal and potentially causal relationship between ADA status, development, and outcome occurrence. With regard to methodology, although the use of ELISA for ADA detection was almost universal (inhibition and neutralizing assays were only used where these were the specific study focus), there was marked variation in sampling frequency and timing (e.g. from baseline and study end only to periodic sampling throughout). Consequently, not all studies captured the time from first infusion to detectable ADA or any rise and fall in titer through the treatment course. Furthermore, few studies reported neutralizing antibody status, which limits the ability to explore the clinical significance of the neutralization capacity of ADAs. Despite these limitations, the review highlights important trends and knowledge gaps, and underscores the need for more standardized and prospective investigations.

This synthesis of available evidence remains valuable for informing future research priorities and advancing understanding of ADA-related effects in Fabry disease. None of the publications included in this SLR were specifically designed to investigate the association between ADA status and Fabry disease-related biomarkers or clinical outcomes in patients receiving ERT. Future prospective studies with this objective in mind should be designed to show temporal change with seroconversion, or that ADA-negative patients (matched to ADA-positive patients for mutation type, residual enzyme activity, and severity) have better outcomes. Analyzing these parameters may provide greater insights into causality; insights that could be further enhanced by evaluating interventions aimed at reducing antibody levels or employing other approaches to reverse the observed rate of change. The design of such studies should take into consideration: (1) relevant patient characteristics (prior treatment and its potential implications for baseline ADA status, mutation type, and residual enzyme activity); (2) ADA titer, subclass and neutralizing activity (recognizing that these may fluctuate over time and may warrant reassessment during the course of a study); and (3) use of standard ADA assessment methods at baseline (to help to distinguish between clinical events associated with underlying disease severity and those potentially associated with temporal changes in ADA and seroconversion). In addition to the need for synchronization of ADA assessment protocols and more complete reporting of such protocols, it would provide valuable insights if future studies concurrently measured disease-related biomarkers at defined time points, and if organ-specific outcomes of particular interest were standardized across studies. Collectively, these strategies could generate more robust evidence to clarify the clinical significance of ADAs in Fabry disease and guide optimization of treatment strategies within this context.

## Conclusions

This is the first systematic evaluation of the relationship between ADAs and ERT clinical and subclinical outcomes in patients with Fabry disease. ADA positivity has been linked to IRR occurrence and increased levels of Fabry disease-related biomarkers, suggesting both the possibility of reduced ERT tolerability and diminished ERT activity in ADA-positive patients. Evidence for associations between ADA presence and renal or cardiovascular outcomes was more variable across studies, and it was not feasible to determine from the available evidence whether any observed associations may have been causal or related to disease severity. However, some data sets indicated a potential trend toward more rapid eGFR decline and worsening cardiac parameters in ADA-positive patients. These observations, although not uniformly consistent within the literature, highlight the need for further investigation into the role of ADAs in organ-specific disease progression. It is also notable that the few studies that controlled for confounding variables, in particular disease severity, reported a significant association between ADA positivity and impaired ERT efficacy. Despite heterogeneity within the source literature, this review underscores the importance of standardized ADA assessments and organ-specific outcomes to understand the full clinical impact of ADA formation and to guide more personalized treatment for patients with Fabry disease.

## Electronic supplementary material

Below is the link to the electronic supplementary material.

Supplementary Material 1

## Data Availability

Chiesi will approve or deny data requests from external parties on a case-by-case basis. Chiesi reserves the right to deny requests for any and all legally appropriate reasons. Data requests that risk sharing participant-level data or proprietary information will not be approved.

## Abbreviations

α-Gal A: α-galactosidase A
AA: Agalsidase alfa
AB: Agalsidase beta
ADA: Antidrug antibody
ADR: Adverse drug reaction
AE: Adverse event
CASP: Critical Appraisal Skills Programme
eGFR: Estimated glomerular filtration rate
ERT: Enzyme replacement therapy
ERTi: Enzyme replacement therapy inhibition
ERT-IgG: Enzyme replacement therapy immunoglobulin G
FD: Fabry disease
Gb3: Globotriaosylceramide
HR: Hypersensitivity reaction
IRR: Infusion-related reaction
LVM: Left ventricular mass
Lyso-Gb3: Globotriaosylsphingosine
NAb: Neutralizing antibodies
PA: Pegunigalsidase alfa
RCT: Randomized controlled trial
SLR: Systematic literature review
