# Supplementary material for: Evaluating the relationship between antidrug antibodies and efficacy and safety outcomes in patients with Fabry disease receiving enzyme replacement therapy: a systematic literature review
Source: Orphanet J Rare Dis. 2026 Jul 16;21:297. doi: 10.1186/s13023-026-04438-8 (PMC13504826; doi:10.1186/s13023-026-04438-8)
Supplement: Supplementary file 1 [file 13023_2026_4438_MOESM1_ESM.docx]

**Supplementary Figures and Tables.**

**Supplementary Table 1.** Embase Literature Search Strategy (conducted 12 January 2024, Case Reports Included)

| Search no. | Search terms |
| --- | --- |
| **Disease** |  |
| #1 | ‘fabry disease’/exp OR ((fabry* NEAR/3 (disease* OR syndrome* OR dyslipidosis OR nephropath*)):ti,ab,de) OR ‘alpha galactosidase deficiency syndrome’:ti,ab,de OR ‘alpha-galactosidase-a deficiency’:ti,ab,de OR ‘angiokeratoma corporis diffusum’:ti,ab,de OR ‘angiokeratoma diffusum’:ti,ab,de OR ‘ceramide trihexosidosis’:ti,ab,de OR ‘deficiency of alpha-galactosidase’:ti,ab,de OR ‘diffuse angiokeratoma’:ti,ab,de OR ‘gla deficienc*’:ti,ab,de OR ‘glycosphingolipid lipidosis’:ti,ab,de OR ‘glycosphingolipidosis’:ti,ab,de OR ‘glycosphingolipoidosis’:ti,ab,de OR ‘hereditary dystopic lipidosis’:ti,ab,de OR ‘mckusick 30150’:ti,ab,de OR ‘thesaurismosis lipoidica’:ti,ab,de |
| **Interventions** |  |
| #2 | ‘drug antibody’/exp OR (((‘anti-drug’ OR antidrug OR drug*) NEAR/2 antibod*):ti,ab,de) OR ada:ti,ab,de OR adas:ti,ab,de |
| #3 | ‘agalsidase alfa’/exp OR ‘agalsidase’:ti,ab,de OR ‘drx 005b’:ti,ab,de OR ‘drx005b’:ti,ab,de OR ‘replagal’:ti,ab,de OR ‘smp 536’:ti,ab,de OR ‘smp536’:ti,ab,de OR ‘tak 675’:ti,ab,de OR ‘tak675’:ti,ab,de OR ‘104138-64-9’:rn |
| #4 | ‘agalsidase beta’/exp OR ‘fabagal’:ti,ab,de OR ‘fabrazyme’:ti,ab,de OR ‘isu 303’:ti,ab,de OR ‘isu303’:ti,ab,de OR ‘jr 051’:ti,ab,de OR ‘jr051’:ti,ab,de OR algasidase:ti,ab,de |
| #5 | ‘enzyme replacement’/exp OR ‘enzyme replacement*’:ti,ab,de OR ert:ti,ab,de |
| #6 | ‘pegunigalsidase alfa’/exp OR ‘pegunigalsidase alfa’:ti,ab,de OR ‘chf 6657’:ti,ab,de OR ‘chf6657’:ti,ab,de OR ‘elfabrio’:ti,ab,de OR ‘peg alfa galactosidase’:ti,ab,de OR ‘peg alpha galactosidase’:ti,ab,de OR ‘pegunigalsidase alpha’:ti,ab,de OR ‘pegylated alfa galactosidase’:ti,ab,de OR ‘pegylated alpha galactosidase’:ti,ab,de OR ‘pegylated unigalsidase alfa’:ti,ab,de OR ‘pegylated unigalsidase alpha’:ti,ab,de OR ‘prx 102’:ti,ab,de OR ‘prx102’:ti,ab,de OR ‘1644392-61-9’:rn |
| #7 | #2 OR #3 OR #4 OR #5 OR #6 |
| #8 | #1 AND #7 |
| **Study types** |  |
| #9 | ‘crossover procedure’:de OR ‘double-blind procedure’:de OR ‘randomized controlled trial’:de OR ‘single-blind procedure’:de OR random*:de,ab,ti OR factorial*:de,ab,ti OR crossover*:de,ab,ti OR ((cross NEXT/1 over*):de,ab,ti) OR placebo*:de,ab,ti OR ((doubl* NEAR/1 blind*):de,ab,ti) OR ((singl* NEAR/1 blind*):de,ab,ti) OR assign*:de,ab,ti OR allocat*:de,ab,ti OR volunteer*:de,ab,ti |
| #10 | ‘post hoc analysis’/exp OR ((‘post hoc’ NEAR/2 (comparison* OR test* OR analys*)):ti,ab,de) |
| #11 | ‘pooled analysis’/exp OR ((pool* NEAR/2 analy*):ti,ab,de) |
| #12 | ‘phase 1 clinical trial’/exp OR ‘phase 1’:ti,ab,de OR ‘phase i’:ti,ab,de |
| #13 | ‘prospective study’/exp OR ‘prospective stud*’:ti,ab,de OR ‘prospective method*’:ti,ab,de |
| #14 | ‘retrospective study’/exp OR ((retrospective NEAR/2 (design* OR stud*)):ti,ab,de) OR ‘ex post facto design*’:ti,ab,de |
| #15 | #9 OR #10 OR #11 OR #12 OR #13 OR #14 |
| #16 | #8 AND #15 |
| **Exclusions** |  |
| #17 | ‘animal’/exp NOT ‘human’/exp |
| #18 | comment*:ti OR ‘letter’:it OR ‘editorial’:it OR ‘short survey’:it |
| #19 | ‘epidemiology’/de OR ‘clinical epidemiology’:ti,ab OR ‘confounding factors (epidemiology)’:ti,ab OR ‘controlled before after stud*’:ti,ab OR ‘controlled before and after stud*’:ti,ab OR ‘effect modifier, epidemiologic’:ti,ab OR ‘effect modifiers (epidemiology)’:ti,ab OR ‘effect modifiers (psychology)’:ti,ab OR ‘environmental epidemiology’:ti,ab OR ‘epidemiologic confounding factors’:ti,ab OR ‘epidemiologic effect modifier’:ti,ab OR ((epidemiologic* NEAR/2 (research* OR stud* OR surve* OR factor* OR method*)):ti,ab) OR ‘epidemiometry’:ti,ab OR ‘historically controlled study’:ti,ab OR ‘interrupted time series anal*’:ti,ab OR ‘precipitating factor*’:ti,ab OR ‘sampling stud*’:ti,ab OR ((ecologic* NEAR/2 (stud* OR research*)):ti,ab,de) |
| #20 | (prognostic NEAR/2 (stud* OR research*)):ti,ab,de |
| #21 | (consensus NEAR/2 (report* OR statement*)):ti,ab,de |
| #22 | ((nonsystematic OR ‘non systematic’) NEAR/2 (review* OR research* OR method*)):ti,ab,de |
| #23 | ‘questionnaire’/exp OR questionnaire*:ti,ab,de OR survey*:ti,ab,de |
| #24 | ‘preclinical study’/exp OR (((‘non clinical’ OR nonclinical OR preclinical OR ‘pre clinical’) NEAR/2 (stud* OR development OR phase* OR ‘drug evaluation*’)):ti,ab,de) |
| **Total** |  |
| #25 | #16 NOT (#17 OR #18 OR #19 OR #20 OR #21 OR #22 OR #23 OR #24) |
| #26 | #25 AND ([article]/lim OR [article in press]/lim OR [erratum]/lim OR [review]/lim) |
| #27 | #25 AND ([conference abstract]/lim OR [conference paper]/lim OR [conference review]/lim) AND [12-1-2022]/sd AND [2022-2024]/py |
| #28 | #26 OR #27 |

**Supplementary Table 2.** PubMed Literature Search Strategy (conducted 12 January 2024, Case Reports Included)

| Search no. | Search terms |
| --- | --- |
| **Disease** |  |
| #1 | “Fabry Disease”[Mesh] OR (fabry[Title/Abstract] AND (disease*[Text Word] OR syndrome*[Text Word] OR dyslipidosis[Text Word] OR nephropath*[Text Word])) OR “alpha galactosidase deficiency syndrome”[Text Word] OR “alpha-galactosidase-a deficiency”[Text Word] OR “angiokeratoma corporis diffusum”[Text Word] OR “angiokeratoma diffusum”[Text Word] OR “ceramide trihexosidosis”[Text Word] OR “deficiency of alpha-galactosidase”[Text Word] OR “diffuse angiokeratoma”[Text Word] OR “gla deficienc*”[Text Word] OR “glycosphingolipid lipidosis”[Text Word] OR “glycosphingolipidosis”[Text Word] OR “glycosphingolipoidosis”[Text Word] OR “hereditary dystopic lipidosis”[Text Word] OR “mckusick 30150”[Text Word] OR “thesaurismosis lipoidica”[Text Word] |
| **Interventions** |  |
| #2 | “anti drug antibod*”[Text Word] OR “antidrug antibod*”[Text Word] OR “drug antibod*”[Text Word] OR “antibody, drug”[Text Word] OR ada[Text Word] OR adas[Text Word] |
| #3 | “agalsidase alfa”[Supplementary Concept] OR “agalsidase”[Text Word] OR “drx 005b”[Text Word] OR “drx005b”[Text Word] OR “replagal”[Text Word] OR “smp 536”[Text Word] OR “smp536”[Text Word] OR “tak 675”[Text Word] OR “tak675”[Text Word] OR “104138-64-9”[rn] |
| #4 | “agalsidase beta”[Supplementary Concept] OR “fabagal”[Text Word] OR “fabrazyme”[Text Word] OR “isu 303”[Text Word] OR “isu303”[Text Word] OR “jr 051”[Text Word] OR “jr051”[Text Word] OR algasidase[Text Word] |
| #5 | “Enzyme Replacement Therapy”[Mesh] OR “enzyme replacement*”[Text Word] OR ert[Text Word] |
| #6 | “pegunigalsidase alfa”[Text Word] OR “chf 6657”[Text Word] OR “chf6657”[Text Word] OR “elfabrio”[Text Word] OR “peg alfa galactosidase”[Text Word] OR “peg alpha galactosidase”[Text Word] OR “pegunigalsidase alpha”[Text Word] OR “pegylated alfa galactosidase”[Text Word] OR “pegylated alpha galactosidase”[Text Word] OR “pegylated unigalsidase alfa”[Text Word] OR “pegylated unigalsidase alpha”[Text Word] OR “prx 102”[Text Word] OR “prx102”[Text Word] OR “1644392-61-9”[rn] |
| #7 | #2 OR #3 OR #4 OR #5 OR #6 |
| #8 | #1 AND #7 |
| **Study types** |  |
| #9 | randomized controlled trial[pt] OR controlled clinical trial[pt] OR randomized[title/abstract] OR placebo[title/abstract] OR clinical trials as topic[mesh:noexp] OR randomly[title/abstract] OR trial[title] |
| #10 | (“post hoc”[Text Word] AND (comparison*[Text Word] OR test*[Text Word] OR analys*[Text Word])) |
| #11 | (pool*[Text Word] AND analy*[Text Word]) |
| #12 | “Clinical Trial, Phase I” [Publication Type] OR “phase 1”[Text Word] OR “phase I”[Text Word] |
| #13 | “Prospective Studies”[Mesh] OR “prospective stud*”[Text Word] OR “prospective method*”[Text Word] |
| #14 | “Retrospective Studies”[Mesh] OR (retrospective[Text Word] AND (design*[Text Word] OR stud*[Text Word])) OR “ex post facto design*”[Text Word] |
| #15 | #9 OR #10 OR #11 OR #12 OR #13 OR #14 |
| #16 | #8 AND #15 |
| **Exclusions** |  |
| #17 | “Animals”[MeSH] NOT “Humans”[MeSH] |
| #18 | “Comment”[Publication Type] OR “Letter”[Publication Type] OR “Editorial”[Publication Type] |
| #19 | “Epidemiologic Studies”[Mesh:NoExp] OR “clinical epidemiology”[Title/Abstract] OR “confounding factors (epidemiology)”[Title/Abstract] OR “controlled before after stud*”[Title/Abstract] OR “controlled before and after stud*”[Title/Abstract] OR “effect modifier, epidemiologic”[Title/Abstract] OR “effect modifiers (epidemiology)”[Title/Abstract] OR “effect modifiers (psychology)”[Title/Abstract] OR “environmental epidemiology”[Title/Abstract] OR “epidemiologic confounding factors”[Title/Abstract] OR “epidemiologic effect modifier”[Title/Abstract] OR “epidemiologic stud*”[Title/Abstract] OR “epidemiologic research*”[Title/Abstract] OR “epidemiologic surve*”[Title/Abstract] OR “epidemiologic factor*”[Title/Abstract] OR “epidemiologic method*”[Title/Abstract] OR “epidemiological stud*”[Title/Abstract] OR “epidemiological research*”[Title/Abstract] OR “epidemiological surve*”[Title/Abstract] OR “epidemiological factor*”[Title/Abstract] OR “epidemiological method*”[Title/Abstract] OR “epidemiometry”[Title/Abstract] OR “historically controlled study”[Title/Abstract] OR “interrupted time series anal*”[Title/Abstract] OR “precipitating factor*”[Title/Abstract] OR “sampling stud*”[Title/Abstract] OR “ecologic stud*”[Text Word] OR “ecologic research*”[Text Word] OR “ecological stud*”[Text Word] OR “ecological research*”[Text Word] |
| #20 | “prognostic stud*”[Text Word] OR “prognostic research*”[Text Word] |
| #21 | “consensus report*”[Text Word] OR “consensus statement*”[Text Word] |
| #22 | “Nonsystematic review*”[Text Word] OR “Nonsystematic research*”[Text Word] OR “Nonsystematic method*”[Text Word] OR “Non systematic review*”[Text Word] OR “Non systematic research*”[Text Word] OR “Non systematic method*”[Text Word] |
| #23 | “Surveys and Questionnaires”[Mesh] OR questionnaire*[Text Word] OR survey*[Text Word] |
| #24 | “non clinical stud*”[Text Word] OR “non clinical development*”[Text Word] OR “non clinical phase*”[Text Word] OR “nonclinical stud*”[Text Word] OR “nonclinical development*”[Text Word] OR “nonclinical phase*”[Text Word] OR “pre clinical stud*”[Text Word] OR “pre clinical development*”[Text Word] OR “pre clinical phase*”[Text Word] OR “pre clinical drug evaluation*”[Text Word] OR “preclinical stud*”[Text Word] OR “preclinical development*”[Text Word] OR “preclinical phase*”[Text Word] OR “preclinical drug evaluation*”[Text Word] |
| **Total** |  |
| #25 | #16 NOT (#17 OR #18 OR #19 OR #20 OR #21 OR #22 OR #23 OR #24) |

**Supplementary Table 3.** Cochrane literature search strategy (conducted 12 January 2024, Case Reports Included)

| Search no. | Search terms |
| --- | --- |
| **Disease** |  |
| #1 | [mh “Fabry Disease”] OR ((fabry* NEAR/3 (disease* OR syndrome* OR dyslipidosis OR nephropath*)):ti,ab,kw) OR “alpha galactosidase deficiency syndrome”:ti,ab,kw OR “alpha galactosidase a deficiency”:ti,ab,kw OR “angiokeratoma corporis diffusum”:ti,ab,kw OR “angiokeratoma diffusum”:ti,ab,kw OR “ceramide trihexosidosis”:ti,ab,kw OR “deficiency of alpha galactosidase”:ti,ab,kw OR “diffuse angiokeratoma”:ti,ab,kw OR (gla NEXT deficienc*):ti,ab,kw OR “glycosphingolipid lipidosis”:ti,ab,kw OR “glycosphingolipidosis”:ti,ab,kw OR “glycosphingolipoidosis”:ti,ab,kw OR “hereditary dystopic lipidosis”:ti,ab,kw OR “mckusick 30150”:ti,ab,kw OR “thesaurismosis lipoidica”:ti,ab,kw |
| **Interventions** |  |
| #2 | (((“anti drug” OR antidrug OR drug*) NEAR/2 antibod*):ti,ab,kw) OR ada:ti,ab,kw OR adas:ti,ab,kw |
| #3 | “agalsidase”:ti,ab,kw OR “drx 005b”:ti,ab,kw OR “drx005b”:ti,ab,kw OR “replagal”:ti,ab,kw OR “smp 536”:ti,ab,kw OR “smp536”:ti,ab,kw OR “tak 675”:ti,ab,kw OR “tak675”:ti,ab,kw OR “104138 64 9”:ti,ab,kw |
| #4 | “fabagal”:ti,ab,kw OR “fabrazyme”:ti,ab,kw OR “isu 303”:ti,ab,kw OR “isu303”:ti,ab,kw OR “jr 051”:ti,ab,kw OR “jr051”:ti,ab,kw OR algasidase:ti,ab,kw |
| #5 | [mh “Enzyme Replacement Therapy”] OR (enzyme NEXT replacement*):ti,ab,kw OR ert:ti,ab,kw |
| #6 | “pegunigalsidase alfa”:ti,ab,kw OR “chf 6657”:ti,ab,kw OR “chf6657”:ti,ab,kw OR “elfabrio”:ti,ab,kw OR “peg alfa galactosidase”:ti,ab,kw OR “peg alpha galactosidase”:ti,ab,kw OR “pegunigalsidase alpha”:ti,ab,kw OR “pegylated alfa galactosidase”:ti,ab,kw OR “pegylated alpha galactosidase”:ti,ab,kw OR “pegylated unigalsidase alfa”:ti,ab,kw OR “pegylated unigalsidase alpha”:ti,ab,kw OR “prx 102”:ti,ab,kw OR “prx102”:ti,ab,kw OR “1644392 61 9”:ti,ab,kw |
| #7 | #2 OR #3 OR #4 OR #5 OR #6 |
| #8 | #1 AND #7 |
| **Study types** |  |
| #9 | [mh ^”Clinical Trials as Topic”] OR trial:ti OR (randomized OR placebo OR randomly):ti,ab OR (“Randomized Controlled Trial” OR “Controlled Clinical Trial”):pt |
| #10 | (“post hoc” NEAR/2 (comparison* OR test* OR analys*)):ti,ab,kw |
| #11 | (pool* NEAR/2 analy*):ti,ab,kw |
| #12 | “Clinical Trial, Phase I”:pt OR “phase 1”:ti,ab,kw OR “phase I”:ti,ab,kw |
| #13 | [mh “Prospective Studies”] OR prospective NEXT stud*:ti,ab,kw OR prospective NEXT method*:ti,ab,kw |
| #14 | [mh “Retrospective Studies”] OR (retrospective NEAR/2 (design* OR stud*)):ti,ab,kw OR “ex post facto” NEXT design*:ti,ab,kw |
| #15 | #9 OR #10 OR #11 OR #12 OR #13 OR #14 |
| #16 | #8 AND #15 |
| **Exclusions** |  |
| #17 | [mh Animals] NOT [mh humans] |
| #18 | (“Comment” OR “Letter” OR “Editorial”):ti |
| #19 | [mh ^”Epidemiologic Studies”] OR “clinical epidemiology”:ti,ab OR (“controlled before after” NEXT stud*):ti,ab OR “controlled before and after”:ti,ab OR (effect NEXT modifier*):ti,ab OR “environmental epidemiology”:ti,ab OR “epidemiologic confounding factors”:ti,ab OR ((epidemiologic* NEAR/2 (research* OR stud* OR surve* OR factor* OR method*)):ti,ab) OR “epidemiometry”:ti,ab OR “historically controlled study”:ti,ab OR (“interrupted time series” NEXT anal*):ti,ab OR (precipitating NEXT factor*):ti,ab OR (sampling NEXT stud*):ti,ab OR ((ecologic* NEAR/2 (stud* OR research*)):ti,ab,kw) |
| #20 | (prognostic NEAR/2 (stud* OR research*)):ti,ab,kw |
| #21 | (consensus NEAR/2 (report* OR statement*)):ti,ab,kw |
| #22 | ((nonsystematic OR “non systematic”) NEAR/2 (review* OR research* OR method*)):ti,ab,kw |
| #23 | [mh “Surveys and Questionnaires”] OR questionnaire*:ti,ab,kw OR survey*:ti,ab,kw |
| #24 | (((“non clinical” OR nonclinical OR preclinical OR “pre clinical”) NEAR/2 (stud* OR development OR phase* OR “drug evaluation” OR “drug evaluations”)):ti,ab,kw) |
| **Total** |  |
| #25 | #16 NOT (#17 OR #18 OR #19 OR #20 OR #21 OR #22 OR #23 OR #24) |
| #26 | #25 NOT “conference proceeding”:pt |
| #27 | #25 AND “conference proceeding”:pt  with Publication Year from 2022 to 2024, with Cochrane Library publication date from Jan 2022 to present, in Trials |
| #28 | #26 OR #27  CDSR: 1  CENTRAL: 63 |

**Supplementary Table 4.** Internet search summary

| **Website** | **Congress year** | **Date of search** |
| --- | --- | --- |
| **Congress** |  |  |
| Fabry Disease Update  [www.fabryupdate.com](http://www.fabryupdate.com) | 2024 | 16 January 2024 |
|  | 2023 | 16 January 2024 |
|  | 2022 | 16 January 2024 |
|  | 2021 | 16 January 2024 |
| Lysosomal Disease Network World Symposium  <https://worldsymposia.org> | 2024 | 16 January 2024 |
|  | 2023 | 16 January 2024 |
|  | 2022 | 16 January 2024 |
|  | 2021 | 16 January 2024 |
| International Congress of Inborn Errors of Metabolism  <https://iciem2021.com.au> | 2024 | 16 January 2024 |
|  | 2023 | 16 January 2024 |
|  | 2022 | 16 January 2024 |
|  | 2021 | 16 January 2024 |
| American Society of Nephrology Kidney Week  [www.asn-online.org/education/kidneyweek](http://www.asn-online.org/education/kidneyweek) | 2024 | 16 January 2024 |
|  | 2023 | 16 January 2024 |
|  | 2022 | 16 January 2024 |
|  | 2021 | 16 January 2024 |
| **Health technology organizations** |  |  |
| National Institute for Health and Care Excellence  <https://www.nice.org.uk/> | NA | 22 January 2024 |
| United States Food and Drug Administration  <https://www.fda.gov/> | NA | 22 January 2024 |
|  | NA | 22 January 2024 |
|  | NA | 22 January 2024 |
| Scottish Medicines Consortium  <https://www.scottishmedicines.org.uk/> | NA | 22 January 2024 |
| Canadian Agency for Drugs and Technologies in Health  <https://www.cadth.ca/> | NA | 22 January 2024 |
|  | NA | 22 January 2024 |
|  | NA | 22 January 2024 |
| **Trial registry** |  |  |
| ClinicalTrials.gov [www.clinicaltrials.gov](http://www.clinicaltrials.gov) | NA | 17 January 2024 |
| ICTRP | NA | 17 January 2024 |

ICTRP, International Clinical Trials Registry Platform; NA, not applicable.

**Supplementary Table 5.** Hand search summary

Citations from systematic literature review and meta-analyses (listed below) were manually searched to identify articles of potential relevance.

**Systematic review/meta analyses included in hand searches citation**

Orsborne C, Black N, Naish JH, Woolfson P, Reid AB, Schmitt M, et al. Disease-specific therapy for the treatment of the cardiovascular manifestations of Fabry disease: a systematic review. Heart. 2024;110(1):19-26.

Lee CL, Lin SP, Niu DM, Lin HY. Fabry disease and the effectiveness of enzyme replacement therapy (ERT) in left ventricular hypertrophy (LVH) improvement: a review and meta-analysis. Int J Med Sci. 2022;19(1):126-31.

Ortiz A, Kanters S, Hamed A, DasMahapatra P, Poggio E, Maski M, et al. Agalsidase beta treatment slows estimated glomerular filtration rate loss in classic Fabry disease patients: Results from an individual patient data meta-analysis. Clin Kidney J. 2021;14(4):1136-46.

Elliott PM, Germain DP, Hilz MJ, Spada M, Wanner C, Falissard B. Why systematic literature reviews in Fabry disease should include all published evidence. Eur J Med Genet. 2019;62(10). doi:10.1016/j.ejmg.2019.103702

Sheng S, Wu L, Nalleballe K, Sharma R, Brown A, Ranabothu S, et al. Fabry’s disease and stroke: Effectiveness of enzyme replacement therapy (ERT) in stroke prevention, a review with meta-analysis. J Clin Neurosci. 2019;65:83-6.

Germain DP, Arad M, Burlina A, Elliott PM, Falissard B, Feldt-Rasmussen U, et al. The effect of enzyme replacement therapy on clinical outcomes in female patients with Fabry disease – a systematic literature review by a European panel of experts. Mol Genet Metab. 2019;126(3):224-35.

El Dib R, Gomaa H, Ortiz A, Politei J, Kapoor A, Barreto F. Enzyme replacement therapy for Anderson-Fabry disease: a complementary overview of a Cochrane publication through a linear regression and a pooled analysis of proportions from cohort studies. PLoS One. 2017;12(3). doi:10.1371/journal.pone.0173358.

El Dib R, Gomaa H, Carvalho RP, Camargo SE, Bazan R, Barretti P, et al. Enzyme replacement therapy for Anderson-Fabry disease. Cochrane Database Syst Rev. 2016 Jul 25;7(7):cd006663.

Rombach SM, Smid BE, Linthorst GE, Dijkgraaf MGW, Hollak CEM. Natural course of Fabry disease and the effectiveness of enzyme replacement therapy: a systematic review and meta-analysis: effectiveness of ERT in different disease stages. J Inherit Metab Dis. 2014;37(3):341-52.

NCT. A study of the safety and efficacy of Fabrazyme in patients with Fabry disease.clinicaltrials.gov/show/NCT00074971.

**Supplementary Table 6.** Publication eligibility criteria – inclusion and exclusion criteria used for screening the title and abstract of identified studies (level 1 screening) and the full text (level 2 screening)

| **Criteria** | **Included** | **Excluded** | |
| --- | --- | --- | --- |
| **Level 1 screening** | | | |
| Population | Patients with Fabry disease  Subgroup of interest: patients with Fabry disease with and without antidrug antibodies | Patients without Fabry disease  Nonhuman | |
| Intervention | Enzyme replacement therapy:  Agalsidase alfa  Agalsidase beta  Pegunigalsidase alfa | Studies that do not have an intervention of interest | |
| Study design | Randomized, controlled, prospective clinical trials  Single-arm studies  Prospective observational studies  Retrospective studies  Registry analyses  Database analyses  Post hoc analyses  Pooled analyses  Case series (including ≥ 2 but ≤ 10 patients) ^a^  Systematic reviews (including meta-analyses) ^b^ | Epidemiological and/or ecological studies  Prognostic studies  Commentaries, letters, or editorials (publication type)  Consensus reports  Nonsystematic reviews  Surveys  Preclinical studies  Case reports (n = 1 patient only) ^c^ | |
| Language | English language only | Non-English language | |
| Date | Database searches: no date limit  Conference abstract searches: past 2 years | Conference abstract searches: before 1 January 2021 | |
| **Level 2 screening** | | | |
| Population | Same as Level 1 screening | | Same as Level 1 screening |
| Intervention | Same as Level 1 screening | | Same as Level 1 screening |
| Comparators | Same as Level 1 screening | | Same as Level 1 screening |
| Study design | Same as Level 1 screening | | Systematic reviews and meta-analyses ^a^ |
| Outcomes | To be included in the review, a study must report at least 1 of the outcomes of interest:  Levels (titers, concentration) or presence of:  ADAs  Inhibition of agalsidase alpha or beta, or pegunigalsidase alfa  ERT inhibition  Neutralizing ADAs  Serum-mediated ERT inhibition  And one of the following:  Renal outcomes/events:  eGFR rate (CFB, slope)  Creatinine levels (CFB)  Renal progression (CFB in CKD stage, UACR)  Renal failure  Chronic kidney disease  Renal transplant  Cardiovascular disease:  Left ventricular mass index  Fibrosis  Cardiovascular outcomes measured by:  Electrocardiogram  Echography  Cardiac magnetic resonance imaging  Cerebrovascular events:  Stroke  Transient ischemic attack  Patient-reported outcomes:  Pain  Gastrointestinal symptoms  Disease severity (by MSSI)  Gb3 levels (plasma, urine, or tissue [skin or podocytes])  LysoGb3 levels  Safety outcomes:  Overall AEs  Serious AEs  Severe AEs  Adherence  Compliance  Discontinuations overall  Discontinuations due to lack of efficacy/safety reasons  Discontinuations due to other reasons  Specific AEs of special interest  Infusion-related reactions  Availability of pharmacokinetic outcomes will be noted in the data-extraction file (e.g., AUC figures, ELISA figures, and serum-mediated inhibition assays)  Other outcomes reporting a relationship between ADAs and Fabry disease will be included at level 2 screening and noted in the data-extraction file ^b^ | | Studies that do not report at least 1 of the outcomes of interest |
| Language | Same as Level 1 screening | | Same as Level 1 screening |
| Date | Same as Level 1 screening | | Same as Level 1 screening |

^a^ Systematic reviews were included at level 1 screening, used for identification of primary studies, and then excluded at level 2 screening.

^b^ Studies reporting a relationship between ADAs and outcomes relevant to Fabry disease were included at level 2 screening and noted in the data-extraction file but not extracted. A comment was made noting the outcome and relationship reported for these studies to account for outcomes not listed in Table 2.

^c^ Case series, including equal to or greater than 2 patients but equal to or less than 10 patients, were included in this SLR because under certain circumstances, such as in rare diseases or preliminary reports of newer drugs, some case series may contribute relevant knowledge that would be informative.

ADA, antidrug antibody; AE, adverse event; AUC, area under the curve; CFB, change from baseline; CKD, chronic kidney disease; eGFR, estimated glomerular filtration; ELISA, enzyme linked immuno-sorbent assay; ERT, enzyme replacement therapy; Gb3, globotriaosylceramide; lysoGb3, globotriaosylsphingosine; MSSI, Mainz Severity Score Index; UACR, urine albumin-to-creatinine ratio.

Note: If it is unclear whether a study met any criterion during the level 1 screening process, the study was progressed to full-text screening to confirm its inclusion in the review.

**Supplementary Table 7**. Quality Assessments of Included Clinical Trials: NICE Checklist[1]

| Vedder 2007[2] | | | | |
| --- | --- | --- | --- | --- |
| Study question | | How is the question addressed in the study? | | Grade^a^ |
| Was randomization carried out appropriately? | | Authors set up a blinded permuted block randomization with a block-size of four. Severely affected and less severely affected patients were randomized separately (i.e., a different set of randomization blocks was applied in both groups) to obtain an equal distribution of disease characteristics. | | Yes |
| Was the concealment of treatment allocation adequate? | | Each block consisted of four envelopes that contained a paper that stated agalsidase alfa or beta. The envelopes were numbered consecutively (randomization number 1 through 4, 5 through 8, etc). These envelopes were generated and checked by 2 persons, who were not involved in obtaining informed consent. | | Yes |
| Were the groups similar at the outset of the study in terms of prognostic factors, for example, severity of disease? | | Severely affected and less severely affected patients were randomized separately (i.e., a different set of randomization blocks was applied in both groups) | | Yes |
| Were the care providers, participants, and outcome assessors blind to treatment allocation? If any of these people were not blinded, what might be the likely impact on the risk of bias (for each outcome)? | | Blinding of the enzyme products was not feasible, since the quality and storage life after rebottling could not be guaranteed. Authors set up a blinded permuted block randomization with a block-size of four. Each block consisted of four envelopes that contained a paper that stated agalsidase alfa or beta | | Not clear |
| Were there any unexpected imbalances in dropouts between groups? If so, were they explained or adjusted for? | | One male patient did not meet the inclusion criteria due to a renal transplant. Eight patients chose to be treated with agalsidase beta at 1.0 mg/kg, either because family members already received this treatment (3 cases), or for other unrelated reasons. These patients did not differ with respect to disease severity from the patients participating in the study. | | Yes |
| Is there any evidence to suggest that the authors measured more outcomes than they reported? | |  | | No |
| Did the analysis include an intention-to-treat analysis? If so, was this appropriate and were appropriate methods used to account for missing data? | |  | | No |
| Wallace 2024[3] | | | | |
| Study question | How is the question addressed in the study? | | Grade^a^ | |
| Was randomization carried out appropriately? | Patients were randomly assigned using a fixed block randomization list (52 blocks of 3 per stratum) stratified at baseline by UPCR incorporated into a Target e*CRF system that generated patient randomization ID numbers once patient eligibility was confirmed by the Protalix medical director. | | Yes | |
| Was the concealment of treatment allocation adequate? | Most persons involved in the study and patients were blinded to the treatment assignment. The pharmacist preparing the study drug was given unblinded access to the e*CRF system and recorded information regarding the prepared dose for each patient. | | Yes | |
| Were the groups similar at the outset of the study in terms of prognostic factors, for example, severity of disease? | Baseline characteristics were similar across arms | | Yes | |
| Were the care providers, participants, and outcome assessors blind to treatment allocation? If any of these people were not blinded, what might be the likely impact on the risk of bias (for each outcome)? | Most persons involved in the study and patients were blinded to the treatment assignment. The pharmacist preparing the study drug was given unblinded access to the e*CRF system and recorded information regarding the prepared dose for each patient. Drug accountability staff were unblinded to treatment. | | Not clear | |
| Were there any unexpected imbalances in dropouts between groups? If so, were they explained or adjusted for? | 78 patients randomized and 77 exposed to treatment | | No | |
| Is there any evidence to suggest that the authors measured more outcomes than they reported? |  | | No | |
| Did the analysis include an intention-to-treat analysis? If so, was this appropriate and were appropriate methods used to account for missing data? | Intent-to-treat (ITT) population was the main set for efficacy analyses and included all randomly assigned patients who received at least one dose (including partial doses). Per protocol (PP) population included ITT patients who completed 24 months of treatment, with ≥80% compliance and no major protocol violations potentially affecting the primary endpoint.  If a patient was missing a value at baseline for one or more of the subgroups, the classification was based on the value at screening. | | Yes | |

^a^Grade: yes/no/not clear/NA

ITT, intent to treat; NA, not applicable; NICE, National Institute for Health and Care Excellence; UPCR, Urine Protein Creatinine Ratio.

**Supplementary Table 8**. Quality Assessments of Included Nonrandomized and Noncontrolled Studies: CASP Checklist[4]

| Lenders 2021[5] | | |
| --- | --- | --- |
| Study question | How is the question addressed in the study? | Grade^a^ |
| Was the cohort recruited in an acceptable way? | 89 patients with genetically confirmed FD from three German Fabry centers (Berlin, Münster and Würzburg) were initially recruited and followed in a prospective observational study. Based on the observational design, patients were not randomized | Yes |
| Was the exposure accurately measured to minimize bias? | Patients who had received agalsidase beta (1.0 mg/kg) for >1 year, which were assigned to continue this treatment (agalsidase-beta, regular-dose group; received a reduced dose of agalsidase-beta and subsequent switch to agalsidase-alfa (0.2 mg/kg) or a direct switch to 0.2 mg/kg agalsidase-alfa (switch group); or were re-switched to agalsidase-beta after receiving agalsidase-alfa for 12 months (re-switch group). The first follow-up of patients within the re-switch group was performed before the re-switch to agalsidase-beta (1.0 mg/kg e.o.w.). The first follow-up of patients within the switch group was adapted to the re-switch group to get comparable observational periods. Serum samples for neutralizing anti-drug antibodies determination were available for at least 38 male patients. 39.5% of tested males were inhibition-positive according to definitions from recent literature. | Yes |
| Was the outcome accurately measured to minimize bias? | To exclude a solely ADA-mediated effect on lyso-Gb3 after a re-switch from agalsidase-alfa to agalsidase-beta, a subset of male patients with available lyso-Gb3 data and ADA status were available was further analyzed. | Yes |
| Have the authors identified all important confounding factors? | Analysis conducted for patients with ADAs but as ADAs could only be determined in a subset of patients, the number of patients for subsequent analyses was further restricted. | Not clear |
| Have the authors taken account of the confounding factors in the design and/or analysis? | Analysis conducted for patients with ADAs but as ADAs could only be determined in a subset of patients, the number of patients for subsequent analyses was further restricted. | Not clear |
| Was the follow-up of patients complete? | The total follow-up was 88 +/- 25 months | Yes |
| How precise (for example, in terms of confidence interval and *P* values) are the results? | CIs are only provided for the outcome of mean decrease of lyso-Gb3 ng/mL, with some larger intervals of −37.2 to −8.2. P-values <0.05 were considered statistically significant | Not clear |
| **Holida 2025[6]** | | |
| **Study question** | **How is the question addressed in the study?** | **Grade^a^** |
| Was the cohort recruited in an acceptable way? | In this phase 3, open-label, international trial, FD patients with documented diagnosis and at least one characteristic disease feature, and at least three years of prior ERT at a stable dose. | Yes |
| Was the exposure accurately measured to minimize bias? | Patients were followed for 52 weeks from a baseline assessment. Blood samples were collected for assessment at centralized laboratories at 12, 24, 40 and 52 weeks. | Yes |
| Was the outcome accurately measured to minimize bias? | Primary (safety) endpoint was assessed to defined terminology (CTCAE). ADAs assessed in designated central laboratory as were PK parameters. | Yes |
| Have the authors identified all important confounding factors? | Limitations in terms of: inherent low number of patients, use of historical, pre baseline data, short treatment duration, etc. extensively discussed | Yes |
| Have the authors taken account of the confounding factors in the design and/or analysis? | Clear description of limitations, no statistical methods (e.g. adjustments, subgroup or sensitivity analyses) conducted specific to the ADA analysis | No |
| Was the follow-up of patients complete? | The only patient not to complete the study withdrew due to a road traffic accident. | Yes |
| How precise (for example, in terms of confidence interval and *P* values) are the results? | Primary endpoint appropriately quantified by number and percentage. Other parameters treated similarly or with SD and IQR. No p values. | Not clear |
| van der Veen 2022[7] | | |
| Study question | How is the question addressed in the study? | Grade^a^ |
| Was the cohort recruited in an acceptable way? | The treated group consists of male cFD patients who participated in the FIELD study, an RCT. For the untreated group, authors conducted a search in the clinical database at the Amsterdam UMC for patients with the same sex and phenotype as the treated patients. | Yes |
| Was the exposure accurately measured to minimize bias? | FD patients with a classical disease phenotype (cFD) who started treatment with agalsidase-beta in childhood were evaluated after 10 years of treatment, and compared to those of untreated male cFD patients. | Yes |
| Was the outcome accurately measured to minimize bias? | The presence of inhibiting antidrug- antibodies (iADAs) to r-αGAL A activity was measured as described in Wanner (2018) expert consensus statement on therapeutic goals in Fabry disease. Exact test that was used, Spearman's rank was used to assess correlations | Yes |
| Have the authors identified all important confounding factors? | Confounding factors are included in the patient characteristics table but not taken into account for the subgroup analysis of the associations of ADAs | Not clear |
| Have the authors taken account of the confounding factors in the design and/or analysis? | Confounding factors are taken into account in the overall analysis but not taken into account for the subgroup analysis of the associations of ADAs | Not clear |
| Was the follow-up of patients complete? | No follow up information reported | Not clear |
| How precise (for example, in terms of confidence interval and *P* values) are the results? | No CIs provided for the ADA association analysis. P values < 0.05 were considered statistically significant | Not clear |
| Lubanda 2009[8] | | |
| Study question | How is the question addressed in the study? | Grade^a^ |
| Was the cohort recruited in an acceptable way? | The trial was an open-label, single-arm study conducted at four centers in four countries where a total of 21 patients were screened; all 21 enrolled and completed the study between June 2003 and April 2006. | Yes |
| Was the exposure accurately measured to minimize bias? | After baseline assessments, patients received 12 infusions of agalsidase beta at 1.0 mg/kg/2 weeks for 6 months (Weeks 0–23), followed by up to 37 infusions at 0.3 mg/kg/2 weeks for 18 months (Weeks 24–96). Infusions of 1.0 mg/kg were administered at an initial rate of 0.25 mg/minute. | Yes |
| Was the outcome accurately measured to minimize bias? | At baseline and every 6 weeks, serum titers of IgG antibodies to agalsidase beta were determined. Patients were considered seropositive when a positive signal on enzyme-linked immunosorbent assay was confirmed by radioimmunoprecipitation. Antibody titers were reported as the inverse of the highest serum dilution that yielded a positive enzyme-linked immunosorbent assay signal. Efficacy and safety assessments were detailed. For the post hoc analysis, a paired t-test was used to compare the baseline value for urinary GL-3 to the average value during the 1.0 mg/kg and the average value during the 0.3-mg/kg period | Yes |
| Have the authors identified all important confounding factors? | Percentage of patients with zero scores for GL-3 accumulation in kidney cells is reported by cell populations and aGAL mutations for the patients are listed | Yes |
| Have the authors taken account of the confounding factors in the design and/or analysis? | A post hoc analysis of anti-aGAL IgG antibodies was conducted where patients were sub grouped by peak titer values, and, for each subgroup, a paired t-test was used to compare the baseline value for urinary GL-3 to the average value during the 1.0 mg/kg and the average value during the 0.3-mg/kg period. | Yes |
| Was the follow-up of patients complete? | Patient follow up not reported | Not clear |
| How precise (for example, in terms of confidence interval and *P* values) are the results? | No CIs provided for the ADA association analysis | Not clear |
| Wilcox 2012[9] | | |
| Study question | How is the question addressed in the study? | Grade^a^ |
| Was the cohort recruited in an acceptable way? | Registry study. The Fabry Registry began enrolling treated and untreated patients in April 2001. All patients with Fabry disease are eligible to enroll in the Fabry Registry, regardless of age, gender, symptoms, or whether they are receiving ERT from any commercial source. | Yes |
| Was the exposure accurately measured to minimize bias? | To be included in the analyses, patients must have received agalsidase beta as their only source of ERT and must have reported at least 1 post-baseline anti-αGAL IgG assessment. | Yes |
| Was the outcome accurately measured to minimize bias? | Anti-αGAL IgG antibody titers were assessed in diluted serum samples by enzyme-linked immunoabsorbent assay at the Genzyme Clinical Specialty Laboratory. Patients were designated as seronegative if they tested negative for anti-αGAL IgG antibodies each time they were tested. Patients were designated as seroconverted, then tolerized if they had 1 or more negative anti-αGAL IgG tests after previously testing positive and whose most recent assessment was negative. Patients were designated as seroconverted, not tolerized if they had at least 1 positive anti-αGAL IgG test and their most recent assessment was positive.  Student's two-sided tests were used to perform statistical significance testing of plasma GL-3 levels between seronegative and seroconverted patients (a p-value cut-point of an alpha-level of 0.05) | Yes |
| Have the authors identified all important confounding factors? | Baseline characteristics table not provided for patients | Not clear |
| Have the authors taken account of the confounding factors in the design and/or analysis? |  | No |
| Was the follow-up of patients complete? | Patient follow up not reported | Not clear |
| How precise (for example, in terms of confidence interval and *P* values) are the results? | No CIs provided for the ADA association analysis but p-values are statistically significant | Not clear |
| Tsurumi 2021[10] | | |
| Study question | How is the question addressed in the study? | Grade^a^ |
| Was the cohort recruited in an acceptable way? | Two post-authorization safety studies, comprising the Special Drug Use Investigation of agalsidase beta (Registry number: AGAL03004) and Drug Use Investigation of agalsidase beta (Registry number: AGAL02904), were conducted. Patient data were added into case report forms using patient medical records at baseline, and at 6-monthly intervals until the end of the study. | Yes |
| Was the exposure accurately measured to minimize bias? | Two post-authorization safety studies, comprising the Special Drug Use Investigation of agalsidase beta (Registry number: AGAL03004) and Drug Use Investigation of agalsidase beta (Registry number: AGAL02904), were conducted. | Yes |
| Was the outcome accurately measured to minimize bias? | Blood samples were collected for antibody titer measurements and GL-3 levels at baseline and prior to study drug administration every 6 months, until the end of the study. The production of anti-agalsidase beta antibodies (IgG) was determined by the enzyme-linked immunosorbent assay (ELISA) and radioimmunoprecipitation (RIP) assay at the Genzyme Clinical Specialty Laboratory. Definitions for AEs and SAEs given in text, Agalsidase beta-related hypersensitivity reactions were defined according to the investigators’ assessment, with adverse events defined as hypersensitivity reactions where the immune response was exaggerated or inappropriate against an antigen, allergen, or another substance. | Yes |
| Have the authors identified all important confounding factors? | Baseline characteristics table provided and includes the presence of complications and clinical symptoms at baseline | Yes |
| Have the authors taken account of the confounding factors in the design and/or analysis? | Subgroup comparisons were performed for patient background factors (age, disease type, Fabry disease complications, concomitant medications and antibody production). | Yes |
| Was the follow-up of patients complete? | Patient follow up not reported, but the discontinuation reasons were recorded. Across the safety analysis sets, a total of 171 patients discontinued treatment with agalsidase beta. Reasons for discontinuation included: insufficient effect (n=6), adverse events (n=8), death (n=26), transfer to a different hospital (n=32), and other reasons (n=103), including switching to other drugs due to the supply shortage of agalsidase beta that occurred in 2009. | Not clear |
| How precise (for example, in terms of confidence interval and *P* values) are the results? | No CIs provided for the ADA association analysis, but majority of p-values are statistically significant | Not clear |
| Lenders 2018[11] | | |
| Study question | How is the question addressed in the study? | Grade^a^ |
| Was the cohort recruited in an acceptable way? | Open cohort study. Inclusion criteria of this open cohort study were that patients were adult men with at least 6 months of any ERT, and had tested positive for neutralizing ADAs | Yes |
| Was the exposure accurately measured to minimize bias? | Patients had at least 6 months of any ERT. | Yes |
| Was the outcome accurately measured to minimize bias? | Serum samples were collected 30–60 minutes before and 30 minutes after the infusion at the opposite arm. Standard ERT inhibition assays to identify inhibition-positive patients were performed. eGFR was quantified using the CKD Epidemiology Collaboration equation on the basis of serum creatinine. Septum thickness was measured as interventricular septum thickness in diastole (IVSd). eGFR, IVSd, and lyso Gb3 data were assessed at ERT-naïve baseline and at the last available visit (2017–2018 follow-up visit) | Yes |
| Have the authors identified all important confounding factors? | Baseline characteristics table for patients provided with outcome measures at baseline | Yes |
| Have the authors taken account of the confounding factors in the design and/or analysis? | Analysis of confounding variables not reported | Not clear |
| Was the follow-up of patients complete? | Patient follow up not reported | Not clear |
| How precise (for example, in terms of confidence interval and *P* values) are the results? | CIs and p-values provided | Yes |
| **Lenders 2016[12]** | | |
| Study question | How is the question addressed in the study? | Grade^a^ |
| Was the cohort recruited in an acceptable way? | Patients had been consecutively recruited at the Fabry center of the University Hospital Muenster (IFAZ) between 2001 and 2014 | Yes |
| Was the exposure accurately measured to minimize bias? | Serum-mediated ERT inhibition assays were performed using patients’ serum samples of the last visit. | Yes |
| Was the outcome accurately measured to minimize bias? | Multivariate regression analysis to assess the influence of serum-mediated ERT inhibition on cardiac and renal measures | Yes |
| Have the authors identified all important confounding factors? | General differences between ERTi– and ERTi+ men are reported in table 1 | Yes |
| Have the authors taken account of the confounding factors in the design and/or analysis? | The mixed model approach for LVmass and RWT calculations was adjusted for age, duration of ERT, prescription of angiotensin aldosterone system blockers, body weight, systolic and diastolic blood pressure. The mixed model approach for eGFR calculation was adjusted for age, duration of ERT, the prescription of renin angiotensin aldosterone system blockers and diuretics | Yes |
| Was the follow-up of patients complete? | Patient follow up not reported | Not clear |
| How precise (for example, in terms of confidence interval and *P* values) are the results? | CI values not frequently reported. P-values reported for all outcomes however the paper does not provide a level of significance provided for this study. | Not clear |
| **Smid 2013[13]** | | |
| Study question | How is the question addressed in the study? | Grade^a^ |
| Was the cohort recruited in an acceptable way? | Authors reviewed clinical records from July 1999 to October 2011 of ERT treated patients | Yes |
| Was the exposure accurately measured to minimize bias? | Patients were treated with agalsidase alpha 0.2 mg/kg/eow, with agalsidase beta 0.2 mg/kg/eow (as part of a different study) or agalsidase beta 1.0 mg/kg/eow. Due to a worldwide shortage of agalsidase beta some patients switched to a lower dose of agalsidase beta (0.5 or 0.25 mg/kg/eow) or to agalsidase alpha. All Dutch Fabry patients who were treated with ERT either in hospital or at home were included, only excluding those who were treated with ERT before home-therapy was available or who were part of an ongoing clinical trial. | Yes |
| Was the outcome accurately measured to minimize bias? | Initially, serum samples were evaluated for the presence of antiagalsidase IgG antibodies by ELISA and/or for neutralizing activity every three months during the first year of therapy and yearly thereafter for males, and yearly for females. IARs were recorded. For all continuous data group differences were assessed by a Mann–Whitney U test. For nominal or categorical data Pearson Chi-Square test was used. Authors considered p-values significant when p≤0.05, we defined a trend as p≤0.10. | Yes |
| Have the authors identified all important confounding factors? | Patient baseline characteristics are reported in table A | Yes |
| Have the authors taken account of the confounding factors in the design and/or analysis? | Predictive factors for the development of serious infusion associated reactions in males are reported in table C | Yes |
| Was the follow-up of patients complete? | Patient follow up not reported | Not clear |
| How precise (for example, in terms of confidence interval and *P* values) are the results? | No CIs reported but significant p-values and level of significance reported for analysis | Yes |
| **Rombach 2012[14]** | | |
| Study question | How is the question addressed in the study? | Grade^a^ |
| Was the cohort recruited in an acceptable way? | Study reports a cohort of 70 adult Dutch Fabry patients and that Analyses were conducted until December 2010 or August 2010, but not details of recruitment | Not clear |
| Was the exposure accurately measured to minimize bias? | Yearly Gb3, lysoGb3 and antibody assessments were performed. Antibody (AB) analysis was performed by incubating plasma of patients with 1 ng agalsidase alfa or beta (depending on the enzyme preparation the patient was treated with). The amount of plasma needed to neutralize 50% of the enzymatic activity of 1 ng agalsidase alfa or beta was determined by titration. A sample was considered anti-agalsidase IgG negative if a sample demonstrated less than 50% neutralization at 5 ml plasma input | Yes |
| Was the outcome accurately measured to minimize bias? | Blood sampling in EDTA was performed for measurement of Gb3 and lyso-Gb3. For Gb3 analysis in urine, A 50 mL urine sample was obtained from a 24 hour urine collection and stored at -20 degrees C until Gb3 analysis was performed essentially as described for plasma. Spearman’s rho was used for correlation of antibody concentration (titer) and changes in (lyso)Gb3 | Yes |
| Have the authors identified all important confounding factors? | Baseline characteristics of all adult classic patients treated >1 year are reported in Table 1 | Yes |
| Have the authors taken account of the confounding factors in the design and/or analysis? | Repeated measurements were used for analysis of LVMass and renal function and Cox regression to analyze time to first white matter lesions/stroke during ERT and the association with (lyso) Gb3 level. | Yes |
| Was the follow-up of patients complete? | Patients with one year of follow-up are included in analysis | Yes |
| How precise (for example, in terms of confidence interval and *P* values) are the results? | No CIs reported but significant p-values and level of significance reported for analysis | Yes |
| **van Breemen 2011[15]** | | |
| Study question | How is the question addressed in the study? | Grade^a^ |
| Was the cohort recruited in an acceptable way? | Fabry patients from the Academic Medical Center (AMC) and from the Universitätsklinikum Würzburg (UKW) who presented with classical Fabry disease and received a minimum of 12 months of ERT were monitored. Control subjects for Gb3 analysis were healthy volunteers | Yes |
| Was the exposure accurately measured to minimize bias? | Patients received three different ERT treatments: infusions every two weeks with agalsidase alfa at 0.2 mg/kg (n=14; 7 males and 7 females), agalsidase beta at 0.2 mg/kg (n=11; 6 males and 5 females), or agalsidase beta at 1.0 mg/kg (n=18; 9 males and 9 females). All patients had been diagnosed by means of a reduced α-Gal A activity in leukocytes (males) and/or a mutation in the α-Gal A gene (females). | Yes |
| Was the outcome accurately measured to minimize bias? | Serum samples were evaluated for the presence of anti-(α-Gal A) antibodies. To assess neutralizing activity, different volumes of serum were incubated with a standard amount of recombinant α-galactosidase A. Enzyme activity was determined after 10 minutes of incubation at room temperature. The serum dilution that resulted in 50% reduction of the enzyme activity was recorded. Plasma Gb3 and lysoGb3 were determined at t=0, t=3 and t=12months of ERT. If available, lipid concentrations were also measured in plasma specimens obtained after 6 and 9 months of treatment. Quantitation of Gb3 in plasma samples was performed. | Yes |
| Have the authors identified all important confounding factors? | Patient baseline characteristics not reported | Not clear |
| Have the authors taken account of the confounding factors in the design and/or analysis? | No additional analysis methods described | Not clear |
| Was the follow-up of patients complete? | Patient follow up not reported | Not clear |
| How precise (for example, in terms of confidence interval and *P* values) are the results? | No CIs reported but p-values are reported for outcomes and level of significance reported for analysis | Yes |
| **Benichou 2009[16]** | | |
| Study question | How is the question addressed in the study? | Grade^a^ |
| Was the cohort recruited in an acceptable way? | Retrospective analysis of data from patients with Fabry disease included in a Phase 3 or Phase 4 placebo-controlled trial | Yes |
| Was the exposure accurately measured to minimize bias? | Blood samples for assay of anti-aGAL IgG were obtained immediately prior to study infusions every 2 weeks and every 12 weeks during the double- blind period of the Phase 3 and Phase 4 studies, respectively, and approximately every 6 months during the open label extensions of both studies. Sera were screened for anti-aGAL IgG by ELISA | Yes |
| Was the outcome accurately measured to minimize bias? | eGFR slopes: Serum creatinine was measured every 6 months in the Phase 3 studies and every month in the Phase 4 studies, and was used to calculate estimated glomerular filtration rate (eGFR) by the 4-variable equation reported in the Modification of Diet in Renal Disease Study. Plasma GL-3 was assessed at 6- and 3-month intervals in the Phase 3 and Phase 4 studies, respectively. Tandem mass spectrometry was used to measure GL-3 concentrations in the plasma | Yes |
| Have the authors identified all important confounding factors? | Titer subgroup analysis only. For each study cohort, data analyses were performed on only males and on males and females; formal analyses of only females were precluded by small patient numbers | Not clear |
| Have the authors taken account of the confounding factors in the design and/or analysis? | Titer subgroup analysis only | Not clear |
| Was the follow-up of patients complete? | Patient follow up not reported | Not clear |
| How precise (for example, in terms of confidence interval and *P* values) are the results? | No CIs reported. P-values are reported for outcomes, but level of significance not reported for analysis, only reported as significant/non-significant in text | Not clear |
| **Khan 2021[17]** | | |
| Study question | How is the question addressed in the study? | Grade^a^ |
| Was the cohort recruited in an acceptable way? | A phase 3/4, open-label, single-arm, multicenter safety study was conducted in Canadian patients with Fabry disease between August 2011 and September 2017 as a regulatory requirement. Eligible patients had a documented diagnosis of Fabry disease and satisfied current Canadian guidelines for receiving enzyme replacement therapy for Fabry disease. | Yes |
| Was the exposure accurately measured to minimize bias? | Treated with agalsidase-alfa AF. Anti-drug antibodies (ADAs) to agalsidase alfa were detected by immunoglobulin G (IgG) enzyme-linked immunosorbent assay or electrochemiluminescence, and in vitro neutralizing anti-drug antibodies (NAbs) were measured by an enzyme inhibition assay | Yes |
| Was the outcome accurately measured to minimize bias? | Adverse event monitoring and assessment of vital signs were assessed at each dosing visit. ADAs were measured by an enzyme inhibition assay. | Yes |
| Have the authors identified all important confounding factors? | Baseline characteristics are listed, which includes medical history | Yes |
| Have the authors taken account of the confounding factors in the design and/or analysis? | Single-arm trial | No |
| Was the follow-up of patients complete? | 140 (83.8%) completed the study and 27 (16.2%) discontinued prematurely. Reasons for discontinuation were listed | Yes |
| How precise (for example, in terms of confidence interval and *P* values) are the results? | No formal statistical tests were conducted. No p-value | No |
| **Riccio 2021[18]** | | |
| Study question | How is the question addressed in the study? | Grade^a^ |
| Was the cohort recruited in an acceptable way? | All Fabry patients attending the Fabry Center of the Federico II University of Naples between September 2006 and November 2020, both already receiving and treatment – naïve to agalsidase beta at the approved dose, were considered for infusion rate escalation protocol. | Yes |
| Was the exposure accurately measured to minimize bias? | Detailed in infusion rate escalation protocol. The development of neutralizing α-GLA A antibodies (Ab status) was assessed by immunochromatographic (IC) assay. | Yes |
| Was the outcome accurately measured to minimize bias? | All clinical and laboratory data of interest were retrospectively gathered from patient clinical charts and electronic health records. In particular, all data were collected at time of protocol initiation (baseline); development of antibodies was assessed by immunochromatographic (IC) assay, after protocol completion, when patients had already achieved the minimum tolerated duration, according to the method described elsewhere. Patients were monitored for IARs during each ERT infusion: specifically, all symptoms occurring during or up to 4 hr after ERT infusions were considered IARs. | Yes |
| Have the authors identified all important confounding factors? | Patients of the two groups were comparable for age, sex, and agalsidase beta dose, while already treated patients had more classic GLA mutations and lower αGal A activity when compared to naΪve patients. None of the patients had received a kidney transplant or used any corticosteroids during the protocol period. | Yes |
| Have the authors taken account of the confounding factors in the design and/or analysis? | Found a negative correlation between infusion duration and αGal A activity, no correlation with mutation type and agalsidase beta dose | Yes |
| Was the follow-up of patients complete? | A total of 23 patients (43.4%) successfully reached the minimum infusion duration of 90 min (20 patients, 16 following the protocol for the dosage of 50–80 mg and 4 following that for the dosage of 80–105), or 120 min (3 patients receiving 80–105 mg of agalsidase beta), but patient follow up was not reported | Not clear |
| How precise (for example, in terms of confidence interval and *P* values) are the results? | Level of significance for all analyses was p < 0.05 for correlation, no CI | Not clear |
| **van der Veen 2019[19]** | | |
| Study question | How is the question addressed in the study? | Grade^a^ |
| Was the cohort recruited in an acceptable way? | A retrospective study was carried out in 39 male patients with classical FD, treated with either agalsidase- alfa or agalsidase-beta. From the total Fabry patient population followed at the Amsterdam Lysosome Center (SPHINX). | Yes |
| Was the exposure accurately measured to minimize bias? | Treated with either agalsidase- alfa or agalsidase-beta | Yes |
| Was the outcome accurately measured to minimize bias? | During follow up, clinical data, as well as plasma samples, were collected at baseline and at every six months during treatment. In vitro plasma-induced enzyme activity inhibition (inhibition titer), lysoGb3 levels and renal function were determined as part of routine care in the hospital. | Yes |
| Have the authors identified all important confounding factors? | The authors tested baseline differences, used linear model, but it is not clear if they considered confounding factors. | Not clear |
| Have the authors taken account of the confounding factors in the design and/or analysis? | The authors tested baseline differences, used linear model, but it is not clear if they considered confounding factors. | Not clear |
| Was the follow-up of patients complete? | Mean follow up of 10 years, retrospective data | Yes |
| How precise (for example, in terms of confidence interval and *P* values) are the results? | Values <0.05 were considered statistically significant, no CI | Not clear |
| **Arends 2018[20]** | | |
| Study question | How is the question addressed in the study? | Grade^a^ |
| Was the cohort recruited in an acceptable way? | Retrospective data from three European FD centers of excellence (Academic Medical Center (AMC), The Netherlands; Royal Free London NHS Foundation Trust, UK and the University Hospital Wuerzburg, Germany) were merged into one database. For the current analysis, these data were combined with the prospectively collected 8 years follow-up data from patients who newly started ERT (cohort 1b) in the CFD. Patients were included who had a definite FD diagnosis according to previously developed criteria | Yes |
| Was the exposure accurately measured to minimize bias? | Patients were treatment naïve and treated with either agalsidase alfa at a dose of 0.2 mg/kg/ EOW or agalsidase beta at a dose of 1.0 mg/kg/EOW for at least 9 months | Yes |
| Was the outcome accurately measured to minimize bias? | Renal function was evaluated using the eGFR and the amount of protein excretion in urine. Cardiac involvement was assessed by echocardiography. Plasma lysoGb3 levels, as reflection of the accumulation of sphingolipids, were measured at the AMC with tandem mass spectrometry using glycine or isotope-labelled lysoGb3 as internal standard. Antibodies were measured as described in Linthorst (2004) | Yes |
| Have the authors identified all important confounding factors? | Time on ERT, ERT type, age at start of ERT, sex, phenotype, ACEi/ARBs, baseline eGFR, baseline LVMI and/ or baseline lysoGb3 were included as covariates when appropriate | Yes |
| Have the authors taken account of the confounding factors in the design and/or analysis? | Applied propensity score matching in a 1:1 ratio (package: MatchIt) in order to assess if an uneven distribution of covariates could bias the results, by using calipers of width equal to 0.1 SD of the estimated propensity score | Yes |
| Was the follow-up of patients complete? | Median follow-up time of 4.9 (0.8–14.4) years. Discontinuation of treatment (n=15) due to patient preferences or treatment failure, change in dose (n=48) or switch of ERT (n=37) occurred in 100 patients (26%), which resulted in censoring. The shortage of agalsidase beta was the main reason to reduce dose (n=47) or switch to agalsidase alfa (n=17). | Yes |
| How precise (for example, in terms of confidence interval and *P* values) are the results? | P values <0.05 were considered statistically significant. Where appropriate, 95% CI are given. | Yes |
| **Mauhin 2018[21]** | | |
| Study question | How is the question addressed in the study? | Grade^a^ |
| Was the cohort recruited in an acceptable way? | The multicenter cohort FFABRY prospectively gathers clinical data and biological samples from patients with an enzymatic and/or genetic diagnosis of FD. Patients were screened for total anti-agalsidase IgG and IgG subclasses | Yes |
| Was the exposure accurately measured to minimize bias? | Exposed to agalsidase | Yes |
| Was the outcome accurately measured to minimize bias? | Clinical data were prospectively collected through a standardized online form. Blood samples were collected at the time of inclusion | Yes |
| Have the authors identified all important confounding factors? | Using linear regression for the assessment of correlations and analysis of covariance, but it is not clear if confounding factors were considered. | Not clear |
| Have the authors taken account of the confounding factors in the design and/or analysis? | Using linear regression for the assessment of correlations and analysis of covariance, but it is not clear if confounding factors were considered. | Not clear |
| Was the follow-up of patients complete? | Prospective cohort, the author did not mention follow-up time | Not clear |
| How precise (for example, in terms of confidence interval and *P* values) are the results? | Using p-value but the authors did not mention the significant value in the statistical analysis. No CI was used | Not clear |
| Tsukimura 2020[22] | | |
| Study question | How is the question addressed in the study? | Grade^a^ |
| Was the cohort recruited in an acceptable way? | Blood samples were obtained from 97 Japanese Fabry patients who had been treated with recombinant a-Gals for at least six months; data were taken from the patients’ clinicians | Yes |
| Was the exposure accurately measured to minimize bias? | To detect anti-drug antibodies (Immunoglobulin G, IgG) in serum from Fabry patients, ELISA was performed. The serum-mediated a-Gal inhibition test was performed to examine neutralizing activity toward purified recombinant a-Gal by means of a modification of the method of Linthorst et al., using serum samples from the Fabry patients obtained at 6 months after the initiation of ERT. Of the 35 classic Fabry males, twenty-seven were treated with agalsidase alfa (0.2 mg/kg/2 weeks), seven with agalsidase beta (1 mg/kg/2 weeks), and one with both (with agalsidase beta for the first 3 months and then with agalsidase alfa thereafter); and of the 17 later-onset Fabry males, 13 were treated with agalsidase alfa and one with agalsidase beta. As to the treatment for the other three, detailed information could not be obtained from the clinicians and of the 45 Fabry females, 33 were treated with agalsidase alfa and 8 with agalsidase beta. Unfortunately, detailed information of the treatment for the other four was not available. | Yes |
| Was the outcome accurately measured to minimize bias? | The differences among the targeted groups were assessed by means of Welch's t-test, it being taken that there was significant difference if p < 0.05. The correlation coefficient between the targeted groups was calculated using Excel 2016. Lyso-Gb3 in plasma was measured by means of liquid chromatography- tandem mass spectrometry (LC-MS/MS) using stable-isotope  labeled lyso-Gb3 as a standard | Yes |
| Have the authors identified all important confounding factors? | Patient baseline characteristics not reported | Not clear |
| Have the authors taken account of the confounding factors in the design and/or analysis? | Patient baseline characteristics not reported | Not clear |
| Was the follow-up of patients complete? | Patient follow up not reported | Not clear |
| How precise (for example, in terms of confidence interval and *P* values) are the results? | No CIs reported but significant p-values and level of significance reported for analysis | Not clear |
| Vedder 2008[23] | | |
| Study question | How is the question addressed in the study? | Grade^a^ |
| Was the cohort recruited in an acceptable way? | Thirty Fabry patients from the Academic Medical Center (AMC) and 1 patient from the Universitatsklinikum Wurzburg (UKW) who were treated with agalsidase alfa or beta at 0.2 mg/kg for a minimum of 12 months were included in the study. Twenty-nine of the AMC patients had been part of the head-to head study. In addition, 5 patients from the AMC and 16 patients from the UKW who had been receiving agalsidase beta at 1.0 mg/kg for at least 12 months were included. | Yes |
| Was the exposure accurately measured to minimize bias? | Patients treated with agalsidase alfa or beta at 0.2 mg/kg for a minimum of 12 months were included in the study. Serum samples were evaluated for the presence of anti-(α-Gal A) antibodies. To assess neutralizing activity, different volumes of serum were incubated with a standard amount of recombinant α-galactosidase A. Enzyme activity was determined after 10 minutes of incubation at room temperature. The serum dilution that resulted in 50% reduction of the enzyme activity was recorded. | Yes |
| Was the outcome accurately measured to minimize bias? | Plasma GL-3 and urinary GL-3 were determined at 0, 3, 6, 9 and 12months of ERT. Quantitation of GL-3 in samples was performed. | Yes |
| Have the authors identified all important confounding factors? | Baseline characteristics of all patients are reported in Table 1 | Yes |
| Have the authors taken account of the confounding factors in the design and/or analysis? | Subgroup analysis by AB status was performed | Yes |
| Was the follow-up of patients complete? | Patient follow up not reported | Not clear |
| How precise (for example, in terms of confidence interval and *P* values) are the results? | Values <0.05 were considered statistically significant, no CI | Not clear |
| **Ohashi 2007[24]** | | |
| Study question | How is the question addressed in the study? | Grade^a^ |
| Was the cohort recruited in an acceptable way? | Fourteen hemizygote patients with a classic Fabry phenotype were divided into two groups. The seronegative group included patients who had no detectable antibody titer during the study. Patients in the seropositive had any detectable antibody formation demonstrated at least once during the study. Details of patient recruitment are not reported. | Not clear |
| Was the exposure accurately measured to minimize bias? | All patients received 1 mg/kg of agalsidase beta intravenously every two weeks. | Yes |
| Was the outcome accurately measured to minimize bias? | Seroconversion with formation of IgG antibodies to agalsidase beta was determined using an ELISA and was confirmed by a radio immunoprecipitation (RIP) assay. Urinary a-Gal A concentration was measured using a sandwich ELISA method. Measurement of uGL-3 was carried out every 3 months as described elsewhere. | Yes |
| Have the authors identified all important confounding factors? | The authors tested baseline differences, but it is not clear if they considered confounding factors. | Not clear |
| Have the authors taken account of the confounding factors in the design and/or analysis? | The authors tested baseline differences, but it is not clear if they considered confounding factors. | Not clear |
| Was the follow-up of patients complete? | Patient follow up not reported | Not clear |
| How precise (for example, in terms of confidence interval and P values) are the results? | No CIs reported but significant p-values and level of significance reported | Not clear |
| **Linthorst 2004[25]** | | |
| Study question | How is the question addressed in the study? | Grade^a^ |
| Was the cohort recruited in an acceptable way? | Patients that were referred to the authors’ hospital for treatment with enzyme supplementation therapy and completed at least 6 months of treatment were included in this study. | Yes |
| Was the exposure accurately measured to minimize bias? | Patients were treated with biweekly agalsidase alpha (Replagal) at a dose of 0.2 mg/kg or agalsidase beta (Fabrazyme) at a dose of 0.2 mg/kg or 1.0 mg/kg. Agalsidase beta 0.2 mg/kg was given as part of another ongoing study. | Yes |
| Was the outcome accurately measured to minimize bias? | In 11 patients, 24-hour urine was collected before start and after 6 months of treatment for determination of globotriaosylceramide (CTH). From 6 patients, 24-hour urine after 12 months of treatment was also available. The Mann-Whitney U test was used to compare urinary CTH levels between IgG-positive and IgG-negative patients. Wilcoxon test was performed to compare urinary CTH levels in individual patients before and after 6 months of treatment. ELISA was used for the detection of anti–rh-a-Gal A IgG antibodies. | Yes |
| Have the authors identified all important confounding factors? | Baseline characteristics table provided and included side effects and mutations | Yes |
| Have the authors taken account of the confounding factors in the design and/or analysis? | Subgroup comparisons were performed for renal function | Yes |
| Was the follow-up of patients complete? | Patient follow up not reported | Not clear |
| How precise (for example, in terms of confidence interval and *P* values) are the results? | CI values not reported. P-values reported for all outcomes however does not provide a level of statistical significance | Not clear |
| **Linhart 2023[26]** | | |
| Study question | How is the question addressed in the study? | Grade^a^ |
| Was the cohort recruited in an acceptable way? | A phase 3, open-label, switch-over, single-arm, multicenter study was conducted. Adults aged 18–60 years with a documented FD diagnosis and ≥1 FD characteristic feature previously receiving agalsidase alfa (0.2 mg/kg E2W) for ≥2 years were eligible. During a 3-month screening/pre-switch period, patients were evaluated monthly while continuing their previous regimen of agalsidase alfa; eligible patients then switched to 1 mg/kg pegunigalsidase alfa intravenous (IV) infusion E2W for 12 months. | Yes |
| Was the exposure accurately measured to minimize bias? | Pegunigalsidase alfa infusions were administered at 1 mg/kg E2W, at a volume adjusted according to patient weight. Infusions were initially administered over 3 h (0.83 mL/min), and if tolerated well, infusion time was gradually reduced to 1.5 h with investigator and medical monitor approval. | Yes |
| Was the outcome accurately measured to minimize bias? | Presence and titers of anti-pegunigalsidase alfa immunoglobulin G (IgG) antibodies were determined using a validated, direct enzyme-linked immunosorbent assay and in vitro enzymatic activity for neutralizing antibodies. Lyso-Gb3 was analyzed in plasma using lipid extraction followed by ultra-performance liquid chromatography/tandem mass spectrometry (UPLC-MS/MS) at a central laboratory. Plasma Gb3 was also analyzed using an HPLC system with ESI–MS/MS. Blood samples were collected at the initial screening visit, and after 3, 6, 9, and 12 months of pegunigalsidase alfa treatment. eGFR was calculated from the CKD-EPI equation, accounting for sCr concentration, age, and sex | Yes |
| Have the authors identified all important confounding factors? | Baseline characteristics table provided | Yes |
| Have the authors taken account of the confounding factors in the design and/or analysis? | Single-arm trial | No |
| Was the follow-up of patients complete? | 20 (91%) completed the study and 2 (9%) discontinued prematurely. Reasons for discontinuation were listed | Yes |
| How precise (for example, in terms of confidence interval and *P* values) are the results? | CI values not reported. P-values reported for some outcomes however does not provide a level of statistical significance | Not clear |

^a^Grade: yes/no/not clear/NA

ADA, antidrug antibody; CI, confidence interval; CKD-EPI, Chronic Kidney Disease-Epidemiology Collaboration equation; E2W, every 2 weeks; eGFR, estimated glomerular filtration rate; ERT, enzyme replacement therapy; FD, Fabry disease; Gb3, globotriaosylceramide; NA, not applicable; NICE, National Institute for Health and Care Excellence; sCr, serum creatinine.

**Supplementary Figure 1.** Plain language summary (PLS) infographic

**References**

1.	NICE. Single technology appraisal and highly specialised technologies evaluation: user guide for company evidence submission template [<https://www.nice.org.uk/process/pmg24/resources/word-version-of-the-company-evidence-submission-main-submission-user-guide-msword-10957826414>]

2.	Vedder AC, Linthorst GE, Houge G, Groener JE, Ormel EE, Bouma BJ, et al. Treatment of Fabry disease: outcome of a comparative trial with agalsidase alfa or beta at a dose of 0.2 mg/kg. PLoS One. 2007;2:e598.

3.	Wallace EL, Goker-Alpan O, Wilcox WR, Holida M, Bernat J, Longo N, et al. Head-to-head trial of pegunigalsidase alfa versus agalsidase beta in patients with Fabry disease and deteriorating renal function: results from the 2-year randomised phase III BALANCE study. J Med Genet. 2024;61:520–30.

4.	Critical Appraisal Skills Programme. CASP Checklist: CASP Randomised Controlled Trial Checklist. [<https://casp-uk.net/casp-tools-checklists/randomised-controlled-trial-rct-checklist/>]

5.	Lenders M, Brand E. Mechanisms of neutralizing anti-drug antibody formation and clinical relevance on therapeutic efficacy of enzyme replacement therapies in Fabry disease. Drugs. 2021;81:1969-81.

6.	Holida M, Linhart A, Pisani A, Longo N, Eyskens F, Goker-Alpan O, et al. A phase III, open-label clinical trial evaluating pegunigalsidase alfa administered every 4 weeks in adults with Fabry disease previously treated with other enzyme replacement therapies. J Inherit Metab Dis. 2025;48:e12795.

7.	van der Veen SJ, Korver S, Hirsch A, Hollak CEM, Wijburg FA, Brands MM, et al. Early start of enzyme replacement therapy in pediatric male patients with classical Fabry disease is associated with attenuated disease progression. Mol Genet Metab. 2022;135:163–9.

8.	Lubanda JC, Anijalg E, Bzduch V, Thurberg BL, Benichou B, Tylki-Szymanska A. Evaluation of a low dose, after a standard therapeutic dose, of agalsidase beta during enzyme replacement therapy in patients with Fabry disease. Genet Med. 2009;11:256–64.

9.	Wilcox WR, Linthorst GE, Germain DP, Feldt-Rasmussen U, Waldek S, Richards SM, et al. Anti-alpha-galactosidase A antibody response to agalsidase beta treatment: data from the Fabry Registry. Mol Genet Metab. 2012;105:443–9.

10.	Tsurumi M, Suzuki S, Hokugo J, Ueda K. Long-term safety and efficacy of agalsidase beta in Japanese patients with Fabry disease: aggregate data from two post-authorization safety studies. Expert Opin Drug Saf. 2021;20:589–601.

11.	Lenders M, Neusser LP, Rudnicki M, Nordbeck P, Canaan-Kuhl S, Nowak A, et al. Dose-dependent effect of enzyme replacement therapy on neutralizing antidrug antibody titers and clinical outcome in patients with Fabry disease. J Am Soc Nephrol. 2018;29:2879–89.

12.	Lenders M, Stypmann J, Duning T, Schmitz B, Brand SM, Brand E. Serum-mediated inhibition of enzyme replacement therapy in Fabry disease. J Am Soc Nephrol. 2016;27:256–64.

13.	Smid BE, Hoogendijk SL, Wijburg FA, Hollak CE, Linthorst GE. A revised home treatment algorithm for Fabry disease: influence of antibody formation. Mol Genet Metab. 2013;108:132–7.

14.	Rombach SM, Aerts JM, Poorthuis BJ, Groener JE, Donker-Koopman W, Hendriks E, et al. Long-term effect of antibodies against infused alpha-galactosidase A in Fabry disease on plasma and urinary (lyso)Gb3 reduction and treatment outcome. PLoS One. 2012;7:e47805.

15.	van Breemen MJ, Rombach SM, Dekker N, Poorthuis BJ, Linthorst GE, Zwinderman AH, et al. Reduction of elevated plasma globotriaosylsphingosine in patients with classic Fabry disease following enzyme replacement therapy. Biochim Biophys Acta. 2011;1812:70–6.

16.	Benichou B, Goyal S, Sung C, Norfleet AM, O'Brien F. A retrospective analysis of the potential impact of IgG antibodies to agalsidase beta on efficacy during enzyme replacement therapy for Fabry disease. Mol Genet Metab. 2009;96:4–12.

17.	Khan A, Sirrs SM, Bichet DG, Morel CF, Tocoian A, Lan L, et al. The safety of agalsidase alfa enzyme replacement therapy in Canadian patients with Fabry disease following implementation of a bioreactor process. Drugs R D. 2021;21:385–97.

18.	Riccio E, Zanfardino M, Franzese M, Capuano I, Buonanno P, Ferreri L, et al. Stepwise shortening of agalsidase beta infusion duration in Fabry disease: clinical experience with infusion rate escalation protocol. Mol Genet Genomic Med. 2021;9:e1659.

19.	van der Veen SJ, van Kuilenburg ABP, Hollak CEM, Kaijen PHP, Voorberg J, Langeveld M. Antibodies against recombinant alpha-galactosidase A in Fabry disease: subclass analysis and impact on response to treatment. Mol Genet Metab. 2019;126:162–8.

20.	Arends M, Biegstraaten M, Wanner C, Sirrs S, Mehta A, Elliott PM, et al. Agalsidase alfa versus agalsidase beta for the treatment of Fabry disease: an international cohort study. J Med Genet. 2018;55:351–8.

21.	Mauhin W, Lidove O, Amelin D, Lamari F, Caillaud C, Mingozzi F, et al. Deep characterization of the anti-drug antibodies developed in Fabry disease patients, a prospective analysis from the French multicenter cohort FFABRY. Orphanet J Rare Dis. 2018;13:127.

22.	Tsukimura T, Tayama Y, Shiga T, Hirai K, Togawa T, Sakuraba H. Anti-drug antibody formation in Japanese Fabry patients following enzyme replacement therapy. Mol Genet Metab Rep. 2020;25:100650.

23.	Vedder AC, Breunig F, Donker-Koopman WE, Mills K, Young E, Winchester B, et al. Treatment of Fabry disease with different dosing regimens of agalsidase: effects on antibody formation and GL-3. Mol Genet Metab. 2008;94:319–25.

24.	Ohashi T, Sakuma M, Kitagawa T, Suzuki K, Ishige N, Eto Y. Influence of antibody formation on reduction of globotriaosylceramide (GL-3) in urine from Fabry patients during agalsidase beta therapy. Mol Genet Metab. 2007;92:271–3.

25.	Linthorst GE, Hollak CE, Donker-Koopman WE, Strijland A, Aerts JM. Enzyme therapy for Fabry disease: neutralizing antibodies toward agalsidase alpha and beta. Kidney Int. 2004;66:1589–95.

26.	Linhart A, Dostálová G, Nicholls K, West ML, Tøndel C, Jovanovic A, et al. Safety and efficacy of pegunigalsidase alfa in patients with Fabry disease who were previously treated with agalsidase alfa: results from BRIDGE, a phase 3 open-label study. Orphanet J Rare Dis. 2023;18:332.
